# Chronic Restraint Stress Misaligns Corneal Clock via β-Adrenergic and Glucocorticoid Signaling, Altering Epithelial, Neural, Immune, Metabolic States

**DOI:** 10.1167/iovs.66.15.50

**Published:** 2025-12-16

**Authors:** Shuting Xuan, Tingting Yang, Mengru Ba, Wenxiao Zhang, Jingwen Yang, Xiaoting Pei, Di Qi, Dingli Lu, Shenzhen Huang, Zhijie Li

**Affiliations:** 1 Department of Ophthalmology, People's Hospital of Henan University, Henan Provincial People's Hospital, Zhengzhou, Henan, China; 2 Department of Ophthalmology, People's Hospital of Zhengzhou University, Henan Provincial People's Hospital, Zhengzhou, Henan, China; 3 Henan Eye Institute, Henan Eye Hospital, and Henan Key Laboratory of Ophthalmology and Visual Science, Henan Provincial People's Hospital, People's Hospital of Zhengzhou University, People's Hospital of Henan University, Zhengzhou, Henan, China

**Keywords:** chronic psychological stress, corneal circadian rhythm, sympathetic nervous system, hypothalamic–pituitary–adrenal (HPA) axis, RNA sequencing, neuro-immune-metabolic coupling

## Abstract

**Purpose:**

To determine whether chronic restraint stress (RS), a validated model of chronic psychological stress, disrupts the corneal circadian transcriptome and alters epithelial, neural, immune, and metabolic outputs, and to distinguish the relative contributions of β-adrenergic and glucocorticoid signaling.

**Methods:**

Male C57BL/6J mice were subjected to RS for 2 h/day at ZT3 (Zeitgeber Time 3) for 14 consecutive days. Cohorts received either propranolol, metyrapone, or no intervention; non-stressed littermates served as controls. Systemic stress activation was confirmed via plasma levels of adrenocorticotropic hormone, corticosterone, epinephrine, and norepinephrine, along with superior cervical ganglion and adrenal gene expression. Whole corneas were collected every three hours over a 24-hour cycle (ZT0–ZT21) and analyzed by bulk RNA sequencing. Rhythmic transcripts were identified using JTK_CYCLE; differential gene screening was performed using DESeq2. Epithelial mitoses, immune-cell counts, ZO-1/occludin expression, subbasal nerve architecture, and Cochet–Bonnet corneal sensitivity were assessed.

**Results:**

RS induced a broad phase advance and amplitude attenuation of rhythmic genes, with downstream reprogramming of immune, proliferative, and metabolic pathways. Phenotypically, RS increased corneal mechanical sensitivity, reduced neutrophil and γδ T-cell counts, elevated epithelial mitotic activity, and diminished ZO-1/occludin expression, whereas subbasal nerve length remained unaffected. Propranolol partially restored circadian phase alignment and improved epithelial and metabolic parameters; metyrapone preferentially enhanced immune-related gene activity and immune-cell abundance.

**Conclusions:**

RS disrupts corneal circadian output and impairs epithelial, immune, and metabolic homeostasis via β-adrenergic and glucocorticoid mechanisms. Temporal targeting of these neuroendocrine pathways may represent a strategy to mitigate stress-induced ocular-surface dysfunction.

Chronic psychological stress (CPS) is a long-term, centrally mediated stress condition that occurs in the absence of overt physical injury and is now recognized as a systemic disturbance disrupting neuroendocrine, immune, and metabolic homeostasis across multiple organ systems, including the ocular surface.^1^^–^^11^ Among experimental paradigms, RS has been widely validated as a reproducible model for inducing CPS in rodents and for dissecting the downstream mechanisms of chronic stress. A hallmark of CPS is the persistent activation of the sympathetic nervous system (SNS) and the hypothalamic–pituitary–adrenal (HPA) axis, leading to prolonged exposure to catecholamines and glucocorticoids. Although these responses are adaptive during acute stress, their chronic activation becomes maladaptive, producing cumulative physiological wear known as allostatic load.^1^^–^^3^

The stress system and the circadian timing system are closely intertwined. The suprachiasmatic nucleus (SCN) orchestrates daily rhythms in physiology and behavior, whereas rhythmic glucocorticoid secretion—peaking near the onset of the active phase—synchronizes peripheral clocks.^12^^,^^13^ Conversely, stress mediators are capable of phase-shifting peripheral oscillators: glucocorticoids reset circadian gene expression, and adrenergic signaling entrains clock programs in the liver.^14^^,^^15^ Such reciprocal regulation suggests that circadian misalignment may contribute to the pathophysiological consequences of chronic stress. Recent system-wide studies have further highlighted the extensive crosstalk between peripheral clocks and systemic zeitgebers, indicating that certain organs may be particularly vulnerable to neuroendocrine cues under sustained stress exposure.^16^

The cornea represents a unique peripheral tissue with dense sensory innervation, active immune surveillance, and robust circadian control. Approximately one quarter of murine corneal transcripts display rhythmic expression under light–dark conditions, and clock timing governs the renewal and repair of corneal epithelium.^17^^,^^18^ Clinically, circadian and psychological disturbances have been associated with ocular-surface dysfunction: sleep deprivation aggravates corneal inflammation through IL-17 signaling, and in humans, tear-film cortisol levels correlate with anxiety severity and dry-eye indexes.^9^^,^^19^ Moreover, recent ocular studies demonstrate that peripheral clocks in retinal and lacrimal tissues are readily reprogrammed by chronic sleep loss or environmental circadian disruption, resulting in altered gene expression and tissue homeostasis.^20^^,^^21^

In peripheral organs, chronic stress frequently alters circadian timing while leaving SCN oscillations largely intact. In the liver, chronic mild stress re-phases clock and metabolic genes such as *Per1*, *Per2*, and *Arntl*.^22^ Repeated social-defeat stress shifts PER2 rhythms across multiple peripheral tissues in a tissue- and time-dependent manner without affecting the SCN.^23^ Within the brain, chronic stress modifies molecular rhythms, reducing amplitude and altering acrophase in regions such as the hippocampus, where these changes correlate with depression-like behaviors.^24^^,^^25^ Collectively, these findings indicate that sustained activation of the SNS and HPA axis can selectively remodel peripheral clocks and thereby perturb local physiological outputs.

At the mechanistic level, peripheral clocks—including those within ocular tissues—are highly responsive to endocrine and autonomic cues. Glucocorticoids act directly on clock-gene promoters to reset circadian timing, whereas adrenergic signaling modulates the phase and amplitude of peripheral oscillations.^13^^–^^15^^,^^26^ Despite these insights, it remains unclear how concurrent activation of the SNS and HPA axis during CPS cooperatively or differentially affects the corneal circadian clock, and how such temporal reprogramming translates into changes in epithelial renewal, neural activity, immune tone, and metabolic balance. Given its structural transparency, rapid epithelial turnover, and integration of sensory and immune inputs, the cornea provides an advantageous model for exploring stress–circadian interactions at the tissue level.^17^^–^^19^^,^^26^

The present study investigates whether RS misaligns the corneal circadian transcriptome and alters epithelial, neural, immune, and metabolic functions through sustained SNS and HPA activation. Using 24-hour, three-hour-interval bulk RNA sequencing, combined with histological and physiological assessments, and bioinformatic analyses such as phase-set enrichment and time-series clustering, we examined the temporal architecture of corneal gene expression under chronic stress. We further assessed the effects of β-adrenergic blockade and glucocorticoid synthesis inhibition to delineate the distinct roles of sympathetic and glucocorticoid pathways in maintaining corneal rhythmicity and homeostasis. The findings reveal that CPS advances the phase and attenuates the amplitude of corneal circadian programs, disrupting epithelial, neural, immune, and metabolic integrity, and that pharmacologic modulation of SNS and HPA activity partially restores these temporal and functional disturbances.

## Materials and Methods

### Experimental Animals

Male C57BL/6J mice (six to eight weeks old, specific pathogen-free) were purchased from the Model Animal Research Center of Nanjing University (Nanjing, China). This strain was selected for its well-established circadian physiology and sensitivity to stress-induced neuroendocrine responses.^27^^–^^29^ On arrival, mice were acclimatized for two weeks under standardized conditions: individually ventilated cages, temperature 22°C ± 1°C, humidity 55% ± 5%, and a strict 12-hour light/dark cycle with lights on at 07:00 (Zeitgeber time [ZT] 0, ZT0) and off at 19:00 (ZT12). Corncob bedding was changed weekly. Ambient noise was maintained below 60 dB, and animal handling was standardized to minimize stress. Mice had free access to standard rodent chow and autoclaved water.

All experimental protocols adhered strictly to the ARVO Statement for the Use of Animals in Ophthalmic and Vision Research and were approved by the Henan Provincial People's Hospital Animal Care and Use Committee (approval no. HNEECA-2022-20). At study completion, animals were euthanized humanely by anesthesia with 3% isoflurane, followed by cervical dislocation. Death was confirmed by cessation of breathing, heartbeat, and corneal reflex.

### Experimental Design

**Figure 1.**
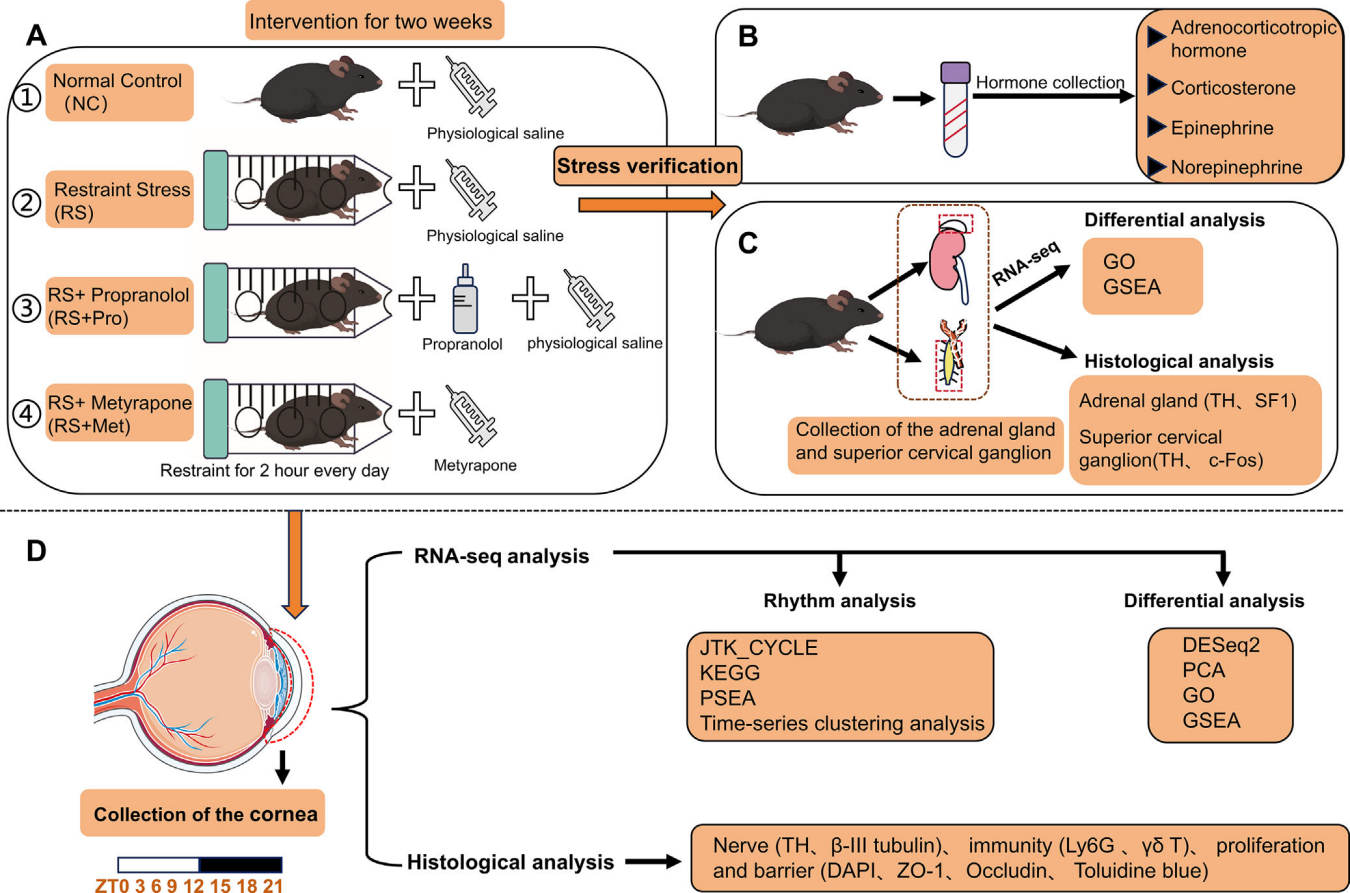
**Schematic overview of the experimental workflow.** (Selected artwork (the "eye") depicted in the figure is derived from or adapted from images provided by Servier Medical Art (Servier; https://smart.servier.com/), licensed under a Creative Commons Attribution 4.0 Unported License). **(A)** Experimental grouping and intervention. Male C57BL/6J mice were randomly divided into four groups: NC, RS, RS + Pro and RS + Met. **(B)** Hormone assessment. Serum ACTH, corticosterone, EPI, and NE levels were quantified by ELISA to evaluate SNS and HPA axis activation. **(C)** Neuroendocrine evaluation. Adrenal glands and SCG were collected for RNA-seq and histological analyses to assess molecular and structural alterations under stress. **(D)** Circadian transcriptomic profiling. On day 14, corneal samples were collected every three hours for RNA-seq. Meanwhile, corneal tissues were collected for immunofluorescence analysis to characterize the disruption of functional and circadian rhythms caused by RS.

The overall experimental design is illustrated in Figure 1. To elucidate whether RS affects the circadian rhythms of the cornea and whether it influences corneal physiological homeostasis by activating the SNS and the HPA axis, mice were randomized into four groups: normal control (NC), restraint stress (RS), RS with propranolol (RS + Pro), and RS with metyrapone (RS + Met). RS mice underwent daily RS (2 h/day) at ZT3-ZT5 for 14 consecutive days. In the RS + Pro group, propranolol (0.5 g/L) was administered via drinking water throughout the stress period. In the RS + Met group, metyrapone (50 mg/kg) was intraperitoneally injected 30 minutes prior to restraint each day. To control for the effect of intraperitoneal injection, all other groups received equivalent volumes of sterile saline (Fig. 1A).

Following the 14-day intervention, physiological and endocrine assessments were conducted. Body weight and fluid intake were recorded to evaluate stress-induced metabolic alterations. Serum was collected and analyzed by enzyme-linked immunosorbent assay to measure adrenocorticotropic hormone (ACTH), corticosterone, epinephrine (EPI), and norepinephrine (NE) levels as indicators of SNS and HPA axis activation (Fig. 1B). To characterize stress-induced neuroendocrine molecular and tissue morphological changes, we collected adrenal glands and superior cervical ganglion (SCG) from mice in each of the four groups for separate RNA-seq and immunofluorescence analyses (Fig. 1C).

For circadian transcriptomic profiling, corneas were collected at three-hour intervals (ZT0, ZT3, ZT6, ZT9, ZT12, ZT15, ZT18, ZT21) across the 24-hour cycle on the final intervention day (three biological replicates per group per time point). Total RNA from corneas was extracted, quality-assessed, and sequenced. Circadian rhythmicity of gene expression was analyzed using the JTK_CYCLE algorithm (period, phase, amplitude). Differentially expressed genes (DEGs) were identified using DESeq2. Functional enrichment and circadian-related analyses—including pathways with Kyoto Encyclopedia of Genes and Genomes (KEGG) enrichment, Gene Ontology (GO) classification, Phase Set Enrichment Analysis (PSEA), time-series clustering, and Gene Set Enrichment Analysis (GSEA)—were performed. Meanwhile, corneal tissues were collected at ZT6 for immunofluorescence staining analysis to comprehensively interpret the biological effects of stress (Fig. 1D).

### RS Model and Pharmacological Interventions

CPS was modeled using a restraint-stress (RS) paradigm as previously validated.^30^ Each day between ZT3 and ZT5, mice were subjected to two hours of standardized physical restraint for 14 consecutive days. Animals were individually placed in 50 mL polypropylene conical restrainers perforated with 0.4 cm ventilation holes and a distal opening to ensure adequate airflow and to prevent physical injury. After each session, mice were returned to their home cages under standard housing conditions.

Pharmacological interventions were implemented in parallel with RS exposure. The RS + Pro group received propranolol (cat# S4076; Selleck Chemicals, Houston, TX, USA) administered ad libitum in drinking water at a concentration of 0.5 g/L, consistent with previously validated regimens.^31^^–^^33^ Because stress can alter water intake, precise mg/kg dosing could not be directly quantified; however, the estimated average consumption under these conditions corresponds to approximately 66–89 mg/kg per day, within the effective range reported in prior studies. The use of a fixed 0.5 g/L concentration was therefore selected to maintain methodological comparability and biological efficacy while acknowledging the inherent limitation of as-desired oral dosing.

The RS + Met group received metyrapone (cat no. S5416; Selleck Chemicals) by intraperitoneal injection at 50 mg/kg, administered 30 minutes before each daily restraint session, to inhibit glucocorticoid synthesis based on previously established effective doses.^34^^,^^35^ Pharmacokinetic data indicate that this dosing regimen provides a pharmacodynamic window sufficient to cover the initial zero- to two-hour phase of RS, during which HPA-axis activation peaks.^36^ To control for injection-related stress, all other groups received equivalent intraperitoneal injections of sterile saline.

### Measurement of Stress-Related Hormones

After the 14-day standardized RS intervention, blood samples were collected at ZT6, corresponding to the midpoint of the inactive (rest) phase for nocturnal mice, when baseline corticosterone and NE levels exhibit relative stability,^37^^–^^39^ thereby enabling sensitive detection of stress-induced hormonal fluctuations (*n* = 6 per group, total *n* = 24; 0.8 ± 0.1 mL per animal). Whole blood was immediately spun in a centrifuge at 3000*g* for 10 minutes at 4°C to isolate serum, which was subsequently used for hormone quantification.

Serum levels of ACTH, corticosterone, EPI, and NE were measured using commercial ELISA kits. The assay kits were obtained from AiFang Biological (Changsha, China), with the following catalog numbers: ACTH (AF2554-A), corticosterone (AF2061-A), EPI (AF2351-A), and NE (AF2533-A). All ELISA procedures were performed strictly according to the manufacturer's instructions.

### Corneal RNA Extraction and RNA-Seq

Corneal tissue collection and RNA extraction were performed as previously described.^17^^,^^19^^,^^40^ After 14 days of intervention, bilateral corneas were collected every three hours over a complete 24-hour circadian cycle on the final day of treatment (ZT0–ZT21, eight time points in total). This design provided uniform temporal coverage for circadian profiling at a three-hour resolution. At each time point, mice were euthanized under isoflurane anesthesia, and paired corneas from both eyes were pooled as one biological replicate (*n* = 3 per time point per group). Immediately after dissection, samples were snap-frozen in liquid nitrogen and stored at −80°C until RNA isolation.

Total RNA was extracted using TRIzol Reagent (Cat no. 15596026; Invitrogen, Thermo Fisher Scientific, Waltham, MA, USA) and purified with the RNeasy Mini Kit (Cat no. 74104; Qiagen, Hilden, Germany). RNA integrity was evaluated with an Agilent 2100 Bioanalyzer, and only samples with an RNA integrity number ≥ 8 were advanced to library preparation.

Sequencing libraries were constructed following Illumina TruSeq protocols and sequenced on an Illumina HiSeq platform (single-end, 50 bp). To ensure adequate transcriptome coverage for rhythmicity analysis, a minimum of 20 million reads per sample was obtained. Raw reads were processed with SOAPnuke (v1.5.6) for adapter trimming and quality filtering. Clean reads were aligned to the mouse reference genome using HISAT (v2.0.4), and gene-level quantification was used for subsequent differential-expression and circadian-rhythm analyses.

### Identification and Pattern Analysis of Circadian Genes

Circadian gene identification and expression profiling were conducted using established methods.^21^^,^^41^^,^^42^ Fragments per kilobase of exon per million reads mapped (FPKM) expression data across eight ZT points (ZT0–ZT21) were imported into R (v4.4.1). Rhythmic genes were identified using the JTK_CYCLE algorithm, which estimates oscillation phase and amplitude, with Bonferroni-adjusted P-values used to assess significance. Genes were classified as: (1) low-expression (FPKM < 0.1), (2) rhythmic (FPKM ≥ 0.1 and adjusted *P* < 0.05), or (3) non-rhythmic (FPKM ≥ 0.1 and adjusted *P* ≥ 0.05).

To evaluate temporal organization at the pathway level, PSEA was performed using KEGG reference sets.^20^^,^^42^ Gene sets were obtained from the Molecular Signatures Database (MSigDB; c2.cp.kegg.v7.2.symbols.gmt), with pathway inclusion criteria limited to sets containing between 10 and 1,000 genes. Pathways showing Benjamini–Hochberg–adjusted *q* values < 0.05 were regarded as significantly enriched, thereby revealing circadian clustering of biological processes across the 24-hour cycle.

Dynamic expression profiles of circadian genes were further analyzed using the Mfuzz package in R (https://bioconductor.org/packages/release/bioc/html/Mfuzz.html).^40^^,^^43^ Fuzzy clustering was performed with a fuzzification parameter of 0.7. Genes were grouped into four expression clusters based on temporal trends. KEGG enrichment analysis was conducted for each cluster to characterize time-specific biological functions.

### Differential Expression Analysis and Functional Enrichment

DEGs were identified using the DESeq2 package (https://bioconductor.org/packages/release/bioc/html/DESeq2.html) in R (v4.4.1). Transcriptomic comparisons were performed between the NC and RS groups at each ZT point. Fold changes (FC) and *P*-values were calculated for each gene. Genes with |log2FC| >0.263 and *P* < 0.05 were considered significantly differentially expressed.

Functional annotation of DEGs included GO biological process categories, conducted via the BGI “Dr. Tom” online platform (https://biosys.bgi.com/#/report/login), following procedures described previously.^44^^,^^45^ Enriched terms with Benjamini–Hochberg adjusted *q* < 0.05 were considered significant.

GSEA was performed using GSEA software (v4.3.3, http://www.broadinstitute.org/gsea/index.jsp). Reference gene sets were obtained from MSigDB (“M5.go.bp.v2022.1.Mm.symbols.gmt”). Significant enrichment was defined as (Normalized Enrichment Score) NES > 1.3 and false discovery rate (FDR) < 0.1.

### Tissue Collection and Immunofluorescence Staining of Adrenal Glands and SCG

To verify systemic neuroendocrine activation in response to RS, SCG and adrenal glands were collected for morphological and molecular analysis at the end of the 14-day intervention, following previously described protocols.^19^^,^^46^ Animals were anesthetized by brief inhalation of 3% isoflurane, consistent with other experimental procedures. Tissues were rapidly dissected under sterile, RNase-free conditions within five minutes after anesthesia. SCG were isolated through a midline cervical incision, gently retracting deep muscles beneath salivary glands and transecting the sternohyoid muscle to expose the carotid artery bifurcation. Bilateral adrenal glands were carefully dissected via midline abdominal incision, removing surrounding fat tissues.

Collected tissues were immediately snap-frozen in liquid nitrogen for RNA-seq or fixed in 4% paraformaldehyde overnight at 4°C for immunofluorescence analysis. Fixed tissues were rinsed thoroughly with PBS, cryoprotected sequentially in 10%, 20%, and 30% sucrose gradients, embedded in optimal cutting temperature compound, rapidly frozen in pre-chilled isopentane at −80°C, and stored until sectioning. Cryosections (10 µm) were prepared using a Leica CM3050 S cryostat (Leica Biosystems, Wetzlar, Germany), mounted onto glass slides, and air-dried.

For immunostaining, sections were blocked for one hour in PBS containing 5% BSA and 0.1% Triton X-100, incubated overnight at 4°C with primary antibodies diluted in blocking buffer (mouse anti-c-Fos, GB12069-100 [Servicebio, Wuhan China]; rabbit anti-TH, GB11181-100 [Servicebio]; rabbit anti-SF1, 18658-1-AP [Proteintech, Rosemont, IL, USA). Following three PBS washes (5 min each), sections were incubated with Alexa Fluor-conjugated secondary antibodies (Alexa Fluor 488 or 594; Invitrogen, Thermo Fisher Scientific) for one hour at room temperature.

Images were acquired using a DeltaVision imaging system (GE-Healthcare, Chicago, IL, USA). Quantitative analysis included the number of c-Fos⁺/TH⁺ double-positive neurons in SCG, the proportion of SF1⁺ cells in the adrenal cortex, and the mean fluorescence intensity of TH⁺ cells in the adrenal medulla, across experimental groups to evaluate changes in sympathetic and endocrine activity. All analyses were performed blinded to group identity.

### Corneal Immunofluorescence Staining and Qualitative Analysis

Immunofluorescence staining of mouse corneas was performed as previously described.^19^^,^^47^ Whole corneas with intact limbus were fixed in 4% paraformaldehyde for one hour at room temperature, followed by thorough washing in PBS. Corneas were subsequently blocked and permeabilized using PBS containing 5% BSA and 0.3% Triton X-100 for one hour at room temperature.

To detect the distribution and density of nerve fibers, whole-mount corneal sections were stained with NL557-conjugated rabbit anti-βIII-tubulin antibody (Cat no. NL1195R; BD Bioscience, Franklin Lakes, NJ, USA) and rabbit anti-tyrosine hydroxylase (TH) antibody (Cat no. CL488-25859; Proteintech). The βIII-tubulin was used to label sensory nerves, whereas TH was used to detect sympathetic innervation. Images were acquired using a DeltaVision Elite imaging system (GE-Healthcare) under a 40 × objective. The length of nerve fibers in the central corneal vortex area was quantified using ImageJ software (National Institutes of Health, https://imagej.nih.gov/ij/) and normalized to unit area.^31^^,^^59^^,^^60^

Immune cell infiltration was assessed using PE-conjugated anti-mouse CD31 (Cat no. 553373; BD Biosciences, San Jose, USA), FITC-conjugated anti-Ly6G (Cat#553127, BD Bioscience), and PE-conjugated anti-mouse γδ-TCR antibody (Cat no. 553178; BD Bioscience). Corneas were radially incised and flat-mounted. Nuclei were counterstained using DAPI (Cat no. G1012; Selleck Chemicals).

Fluorescent images were acquired with a DeltaVision Elite imaging system (GE Healthcare, USA) using consistent exposure settings and fluorescence channels (40 × magnification). Neutrophil counts were performed in eight predefined limbal vascular fields (green fields, Supplementary Fig. 1). γδ-T cells were quantified separately in the epithelial and stromal layers in three limbal-to-central zones (orange fields 1–3, Supplementary Fig. 1). Corneal epithelial cell proliferation was evaluated by identifying DAPI-labeled mitotic figures with paired nuclei (60 × magnification). Nine equidistant regions were defined along two perpendicular corneal axes (orange regions, Supplementary Fig. 1), and mitotic cells were summed across these fields.^19^^,^^40^^,^^48^

Corneal barrier integrity was evaluated by staining for ZO-1 (Cat# CL594-21773, Proteintech) and Occludin (Cat no. CL488-27260; Proteintech). Fluorescence intensity was quantified in standardized corneal regions using ImageJ.

### Assessment of Corneal Nerve Sensitivity and Corneal Barrier Function

Corneal mechanical sensitivity was evaluated using a Cochet-Bonnet aesthesiometer (Model 8630-1490-29; Luneau SAS, Plumeil-le-Gillon, France), following established protocols.^19^^,^^49^ The instrument uses a standardized nylon filament of varying lengths to deliver calibrated mechanical stimuli and determine the tactile threshold of the cornea.

During testing, mice were awake and gently restrained to minimize movement artifacts. The initial filament length was set at 6 cm. If no blink reflex was elicited, the filament was shortened in 5 mm increments, with consistent intervals between each stimulus. At each step, the filament was applied perpendicularly to the central corneal surface. The filament length that first elicited a reproducible blink response was recorded as the mechanical sensitivity threshold.

After the two-week intervention period, corneal barrier integrity was evaluated using fluorescein staining. A 2-µL aliquot of 1% sodium fluorescein solution was instilled into the conjunctival sac of each mouse. After 90 seconds, the ocular surface was gently rinsed with sterile saline solution, and residual fluid was removed using a sterile cotton swab. The cornea was then immediately examined under cobalt blue illumination using a slit-lamp biomicroscope. The extent of fluorescein uptake was documented and graded to assess epithelial permeability.

All tests were conducted in a quiet room with stable lighting and ambient temperature. To reduce variability, all assessments were performed by a single trained examiner at ZT6, ensuring consistency in environmental conditions and procedural handling.

### Statistical Analysis and Software Tools

All data were visualized and analyzed using GraphPad Prism (version 8.0.1; GraphPad Software, La Jolla, CA, USA). Before statistical analysis, data were assessed for normality using the Shapiro-Wilk test. For normally distributed data with homogeneous variances, two-group comparisons were conducted using unpaired Student's *t*-tests. Comparisons among multiple groups were performed using one-way ANOVA, followed by Bonferroni multiple-comparison tests. For non-normally distributed or heteroscedastic data, non-parametric Mann–Whitney U (two groups) or Kruskal–Wallis tests followed by Dunn's multiple comparisons (multiple groups) were used. Statistical significance was set at *P* < 0.05, with adjusted thresholds clearly indicated where multiple comparisons were applied (Bonferroni or Benjamini-Hochberg FDR corrections).

To compare circadian parameters across groups, the Kruskal–Wallis test was used to assess differences in amplitude and median expression levels, with 95% confidence intervals (CIs) estimated accordingly. Differences in peak phase (acrophase) were evaluated using the Watson–Williams circular ANOVA test. For all multiple comparisons, *P*-values were adjusted using the FDR method, and statistical significance was defined as *q* < 0.05.

Heatmaps illustrating transcriptomic data were generated using the pheatmap package in R (version 4.4.1), with Euclidean distances and hierarchical clustering via Ward's method. PCA was conducted using STAMP software (version 2.1.3, https://beikolab.cs.dal.ca/software/STAMP) to assess global transcriptional variation among treatment groups. Phase distributions of circadian genes were visualized using Oriana (version 4.0.1; Kovach Computing Services, Pentraeth, Wales, UK).

## Results

### RS Induces Activation of the SNS and HPA Axis

**Figure 2.**
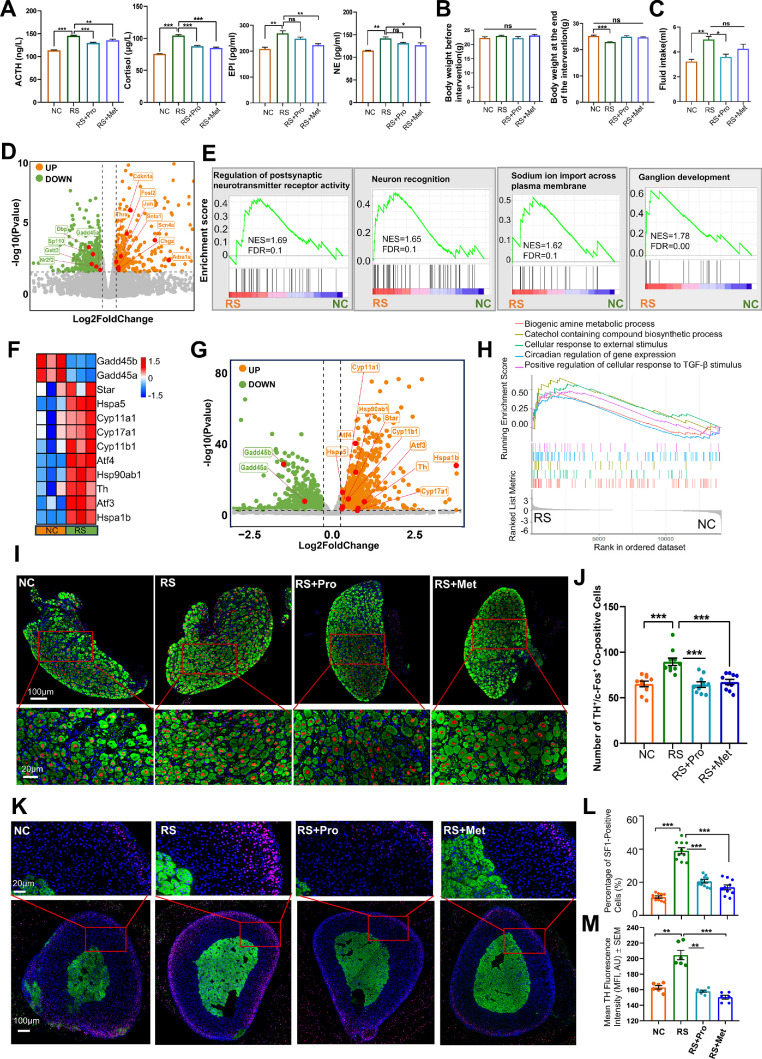
**RS activates the SNS and HPA axis in mice.** **(A)** Serum levels of ACTH, corticosterone, EPI, and NE measured by ELISA in NC, RS, RS + Pro, and RS + Met groups (mean ± SEM, *n* = 6). *P < 0.05, **P < 0.01, ***P < 0.001. **(B)** Body weight measurements before and after 14 days of treatment (mean ± SEM, *n* = 6). ***P < 0.001. **(C)** Average daily water intake of the four groups of mice during the intervention period (mean ± SEM, *n* = 6). *P < 0.05, **P < 0.01. **(D)** Volcano plot showing DEGs in the SCG between RS and NC groups (|log₂FC| > 0.263, *P* < 0.05). *Orange* and *green dots* indicate significantly upregulated and downregulated genes, respectively. Representative genes are labeled. **(E)** GSEA of SCG transcriptomes showing representative pathways significantly enriched in the RS group (*n* = 3). **(F)** Heatmap of Z-score–normalized expression of selected stress- and steroidogenesis-related genes in adrenal tissues from NC and RS groups. **(G)** Volcano plot of DEGs in adrenal tissue between RS and NC groups (|log₂FC| > 0.263, *P* < 0.05). *Orange* and *green dots* indicate significantly upregulated and downregulated genes, respectively. Selected genes are labeled. **(H)** GSEA of adrenal transcriptomes showing representative pathways enriched in the RS group (*n* = 3). **(I)** Representative immunofluorescence images of SCG sections stained for tyrosine hydroxylase (TH, *green*), c-Fos (*red*), and DAPI (*blue*). Upper panels: low magnification (*scale bar*: 100 µm); lower panels: higher magnification (*scale bar*: 20 µm). **(J)** Quantification of TH⁺/c-Fos⁺ double-positive cells in the SCG (mean ± SEM, *n* = 6). ***P < 0.001. **(K)** Representative immunofluorescence images of adrenal sections stained for TH (*green*, medulla), SF1 (*red*, cortex), and DAPI (*blue*). *Lower panels*: whole section view (*scale bar*: 100 µm); upper panels: magnified views (*scale bar*: 20 µm). **(L)** Quantification of SF1⁺ cells in the adrenal cortex as a percentage of total DAPI⁺ nuclei (mean ± SEM, *n* = 6). ***P < 0.001. **(M)** Semi-quantification of TH fluorescence intensity in the adrenal medulla, normalized using ImageJ (mean ± SEM, *n* = 6). **P < 0.01, ***P < 0.001.

To confirm systemic neuroendocrine activation induced by RS, we measured circulating stress-related hormones and examined tissue-specific transcriptomic and protein changes in key neuroendocrine organs. Serum ACTH, corticosterone, EPI and NE were significantly higher in RS mice than in NC (*P* < 0.01), whereas body weight decreased by 9.45% (*P* < 0.001), indicating systemic stress and metabolic load (Figs. 2A, 2B). Moreover, compared with the control group, mice subjected to RS exhibited a significant increase in water intake (Fig. 2C). Pharmacological treatment partially reversed these alterations.

RNA-seq of the SCG revealed 578 upregulated and 545 downregulated genes in RS mice versus NC (|log₂FC| ≥ 0.263, *P* < 0.05; Fig. 2D). Upregulated genes included sympathetic-signaling mediators such as *Snta1, Thra* and *Chga*. GSEA showed significant enrichment of pathways related to sympathetic activation, including “Regulation of postsynaptic neurotransmitter receptor activity” (NES = 1.69, FDR = 0.1) and “Neuron recognition” (NES = 1.65, FDR = 0.1; Fig. 2E).

In adrenal tissue, 1538 genes were upregulated and 1711 downregulated in RS mice compared with controls (|log₂FC| ≥ 0.263, *P* < 0.05; Figs. 2F, 2G). Steroidogenesis-related genes (*Star*, *Cyp11a1*, *Cyp11b1*, *Cyp17a1*) and stress-response genes (*Atf4*, *Hspa5*, *Th*) were prominently induced. GSEA identified significant enrichment of pathways associated with steroidogenesis, catecholamine biosynthesis and cellular stress responses (NES > 1.30, FDR <0.1; Fig. 2H).

Immunofluorescence confirmed these transcriptomic changes. RS mice exhibited a higher proportion of TH⁺/c-Fos⁺ double-positive neurons in the SCG than NC (*P* < 0.001; Figs. 2I, 2J). In the adrenal gland, the number of SF1⁺ cortical cells increased (*P* < 0.001), and TH fluorescence intensity in the medulla was enhanced (*P* < 0.001; Figs. 2K–M). Both propranolol and metyrapone significantly mitigated these tissue-level alterations (*P* < 0.01), indicating that sympathetic and glucocorticoid pathways make distinct yet complementary contributions to RS-induced neuroendocrine activation.

### Chronic Stress Remodels Circadian Transcriptomic Features in the Mouse Cornea

**Figure 3.**
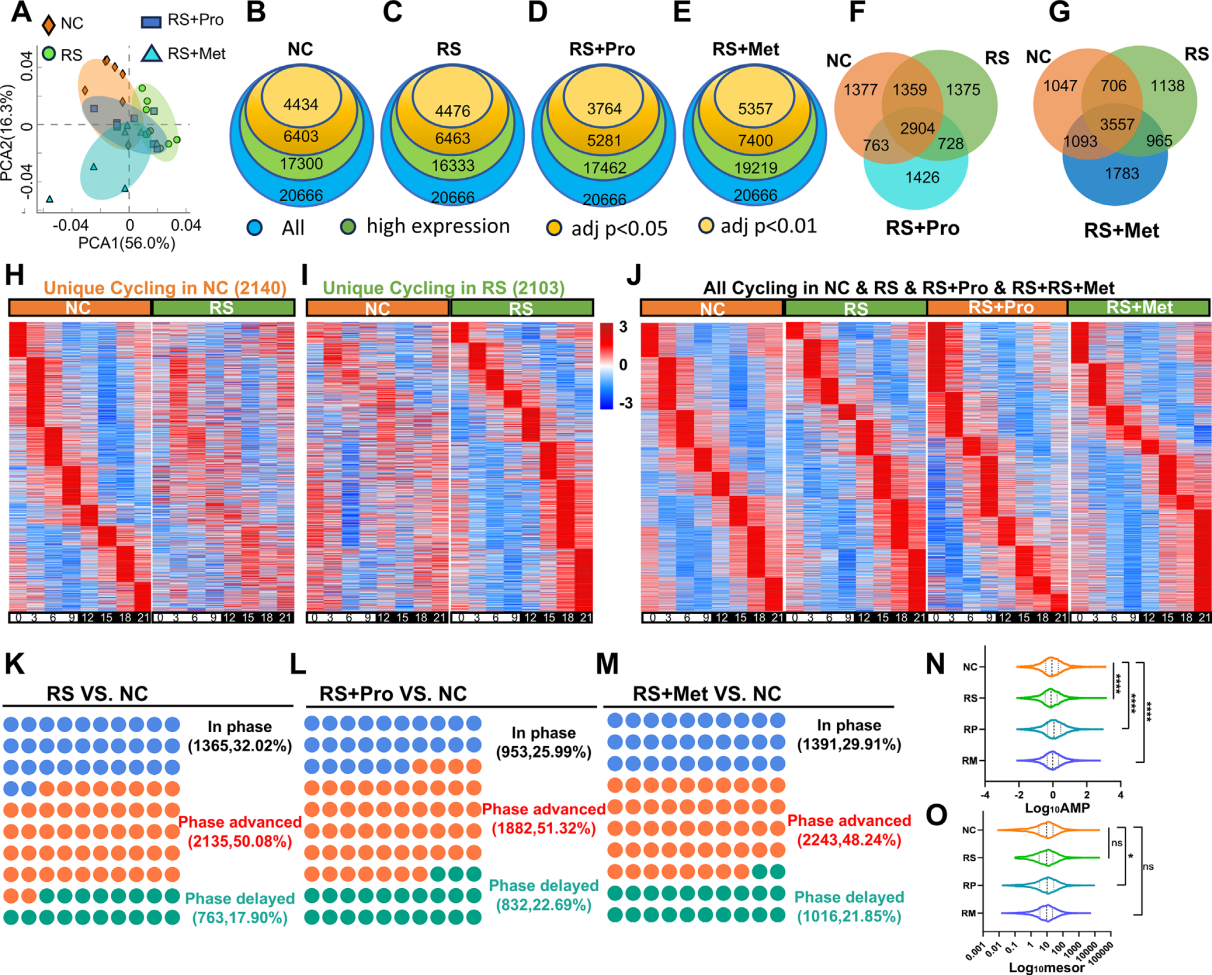
**Circadian transcriptomic features of the mouse cornea under chronic stress and pharmacological intervention.** **(A)** PCA of average gene expression profiles across 8 ZT points (ZT0–ZT21) over 24 hours in the NC, RS, RS+Pro, and RS+Met groups (*n* = 3). **(B–E)** Overlap of expressed genes in each group. *Colored circles* represent total expressed genes (*blue*), highly expressed genes (*green*), and significantly changed genes under different thresholds (*brown*: adj *P* < 0.05; *orange*: adj *P* < 0.01), for NC **(B)**, RS **(C)**, RS + Pro **(D)**, and RS + Met **(E)**. **(F)** Venn diagram showing rhythmic genes shared among NC, RS, and RS + Pro groups. **(G)** Venn diagram showing rhythmic genes shared among NC, RS, and RS+Met groups. **(H)** Heatmap showing the expression of NC-specific rhythmic genes across ZT0–ZT21, compared with the RS group. **(I)** Heatmap showing expression patterns of RS-specific rhythmic genes compared to NC group. **(J)** Heatmap of rhythmic genes detected across all four groups over 24 hours. **(K–M)** Summary of phase shift categories of rhythmic genes relative to NC in RS (K), RS + Pro (L), and RS + Met (M). *Blue*: unchanged phase; *orange*: phase advanced; *green*: phase delayed. **(N)** Violin plots showing the distribution of log₁₀-transformed amplitudes of rhythmic genes in each group. ****Bonferroni-corrected *P* < 0.0001. **(O)** Violin plots showing the distribution of log₁₀-transformed mesor values of rhythmic genes in each group. * Bonferroni-corrected *P* < 0.05.

To evaluate the effects of chronic stress and pharmacological intervention on global transcriptomic dynamics in the mouse cornea, PCA was performed on RNA-seq data collected across eight ZT points from NC, RS, RS + Pro, and RS + Met groups. PCA revealed clear separation among the NC, RS, and RS + Met groups, whereas the RS + Pro group showed partial overlap with the NC group (Fig. 3A), suggesting a potential partial restoration of transcriptomic profiles by propranolol.

Across all groups, a total of 20,666 expressed genes were detected. Using JTK_CYCLE with Bonferroni-adjusted *P* < 0.05, we identified 6403, 6463, 5821, and 7400 circadian-related genes (CRGs) in the NC, RS, RS + Pro, and RS + Met groups, respectively (Figs. 3B–E). Venn diagram analysis revealed that 4263 CRGs were shared between NC and RS groups, whereas 2140 and 2013 CRGs were unique to the NC and RS groups, respectively (Fig. 3F). Comparisons between NC and RS + Pro or RS + Met groups showed 763 and 1093 uniquely overlapping CRGs, respectively (Figs. 3F, 3G), indicating distinct regulatory patterns under the two drug interventions.

Temporal heatmaps showed clear phase-specific expression patterns. CRGs unique to the NC group peaked mainly in the light phase (ZT0-ZT12), whereas CRGs unique to the RS group peaked largely in the dark phase (ZT12-ZT21) (Figs. 3H, 3I). In RS corneas the global CRG profile shifted toward nighttime expression. Propranolol partially restored a daytime-dominant pattern, whereas metyrapone-treated samples retained the nighttime bias observed in RS (Fig. 3J).

Phase-shift analysis showed pronounced circadian reprogramming under RS. Of the CRGs shared with NC, 50.1% were phase-advanced, 17.9% were phase-delayed and 32.0% were unchanged (Fig. 3K). In RS + Pro corneas 51.3% of shared CRGs were advanced, 22.7% delayed and 26.0% unchanged (Fig. 3L). Metyrapone showed a slightly smaller proportion of advanced phase (48.2 %) (Fig. 3M). Overall, phase advance emerged as the predominant circadian response to RS and persisted after pharmacologic intervention.

Analysis of CRG oscillation amplitudes revealed that the RS group exhibited a significant reduction in rhythmic amplitude compared to the NC group (median difference = –0.061; 95% CI, –0.081 to –0.039; Bonferroni-corrected *P* < 0.0001) (Fig. 3N). Although both the RS + Pro and RS + Met groups showed partial recovery, neither intervention fully restored normal amplitude levels. Notably, RS did not significantly alter the overall median expression of CRGs (median difference = –0.109; 95% CI, –0.166 to 0.381; *P* = 0.42), whereas propranolol treatment significantly increased oscillatory output (Fig. 3O).

In summary, RS markedly disrupts the corneal circadian program, inducing widespread phase advancement and dampened amplitude. Propranolol more effectively corrected phase misalignment than metyrapone, indicating that sympathetic activation is the primary contributor to the phase shift. In contrast, full restoration of rhythmic amplitude likely requires broader modulation of neuroendocrine pathways beyond β-adrenergic blockade.

### Chronic Stress Reshapes the Functional Enrichment and Circadian Phase Distribution of Corneal CRGs

**Figure 4.**
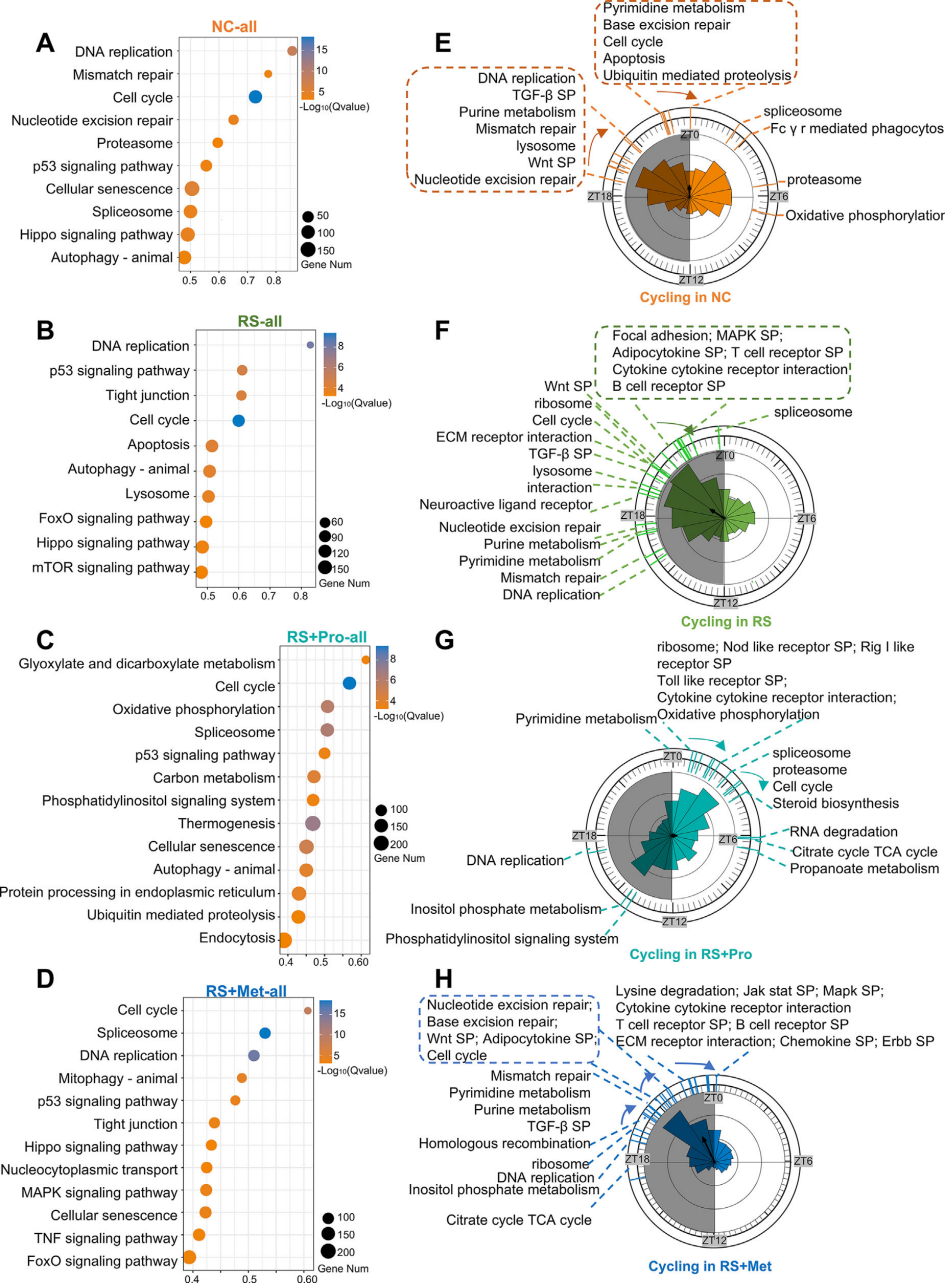
**KEGG pathway analysis and circadian phase distribution of rhythmic gene-enriched pathways in the mouse cornea under chronic stress and pharmacological interventions.** **(A–D)** KEGG pathway enrichment bubble plots showing the top 10 significantly enriched pathways (*q* < 0.05) in the NC **(A)**, RS **(B)**, RS + Pro **(C)**, and RS + Met **(D)** groups. Bubble color indicates −log₁₀ (*q* value), and bubble size reflects the number of genes involved in each pathway. **(E–H)** PSEA of KEGG pathways enriched among rhythmic genes in the NC **(E)**, RS **(F),** RS + Pro **(G)**, and RS + Met **(H)** groups (*q* < 0.05). Polar bar plots depict the circadian phase distribution of rhythmic genes in each condition. *Colored dashed lines* around the outer ring indicate ZT points at which individual pathways are significantly enriched. *Gray-shaded areas* denote the dark phase (ZT12–ZT24).

Having characterized global alterations in circadian phase and amplitude, we next evaluated how these shifts reorganized functional gene networks and pathway enrichment patterns under chronic stress and pharmacologic intervention. Circular phase plots of CRGs revealed significant differences in group mean phase (Watson–Williams test, *P* < 0.0001), accompanied by marked changes in phase timing, vector direction, and vector concentration. The mean phase vectors were as follows: NC, µ = 23:57; RS, µ = 20:07; RS + Pro, µ = 05:52; RS + Met, µ = 22:18. Corresponding vector lengths were NC: *r* = 0.187; RS: *r* = 0.251; RS + Pro: *r* = 0.086; RS + Met: *r* = 0.390 (Figs. 4E–H). Post hoc pairwise comparisons showed that RS advanced the mean phase by approximately 3.84 hours relative to NC (95% CI, 3.49–4.19 hours; Bonferroni-corrected *P* < 0.0001), indicating robust phase misalignment after chronic stress.

To determine how RS alters both the functional landscape and temporal coordination of corneal CRGs, we carried out KEGG enrichment and PSEA. In NC, CRGs were primarily associated with core housekeeping pathways—including DNA replication, mismatch repair and cell-cycle regulation (Fig. 4A). By contrast, CRGs newly recruited in the RS group were enriched for immune activation, proliferative signaling and stress-response pathways, with prominent representation of the FoxO and mTOR cascades (Fig. 4B), indicating a marked functional reprogramming of the circadian transcriptome under stress.

Pharmacological intervention with propranolol shifted the enrichment of CRGs toward metabolic pathways, including glyoxylate and dicarboxylate metabolism and oxidative phosphorylation (Fig. 4C). In contrast, metyrapone maintained a pathway enrichment pattern similar to RS, with sustained dominance of immune-related pathways (Fig. 4D), indicating differential regulatory effects between the two treatments on circadian gene function.

PSEA confirmed a marked temporal reorganization of pathway activity. In controls, enriched pathways clustered at two windows—ZT18-ZT21 and ZT0-ZT6 (Fig. 4E). RS shifted this pattern toward the late-night interval (ZT15-ZT22.5) and was dominated by immune-related signaling (Fig. 4F). Propranolol advanced the peak to the early light phase (ZT1.5-ZT4.5) and substantially reduced the fraction of immune pathways (Fig. 4G). By contrast, metyrapone retained the nighttime bias (ZT18–ZT0) observed in the RS group (Fig. 4H).

Quantitative comparison of the two pharmacological interventions revealed distinct patterns of circadian and functional recovery. In PSEA, the RS group exhibited pathway enrichment predominantly during the late night (ZT15–ZT22.5), characterized by an overrepresentation of immune-related signaling cascades. Propranolol treatment markedly advanced these enrichment peaks to the early light phase (ZT1.5–ZT4.5) and substantially reduced the relative proportion of immune pathways, indicating a corrective effect on both phase timing and immune dysregulation. In contrast, metyrapone intervention largely maintained the RS-like nocturnal bias, with limited phase realignment and continued dominance of immune-pathway enrichment. This divergence is clearly visualized in the polar plots, where the propranolol-treated group (Fig. 4G) displays a restored light-phase clustering, whereas the metyrapone group (Fig. 4H) retains the stress-induced night-phase accumulation.

### Dynamic Expression Patterns and Temporal Pathway Enrichment of CRGs

**Figure 5.**
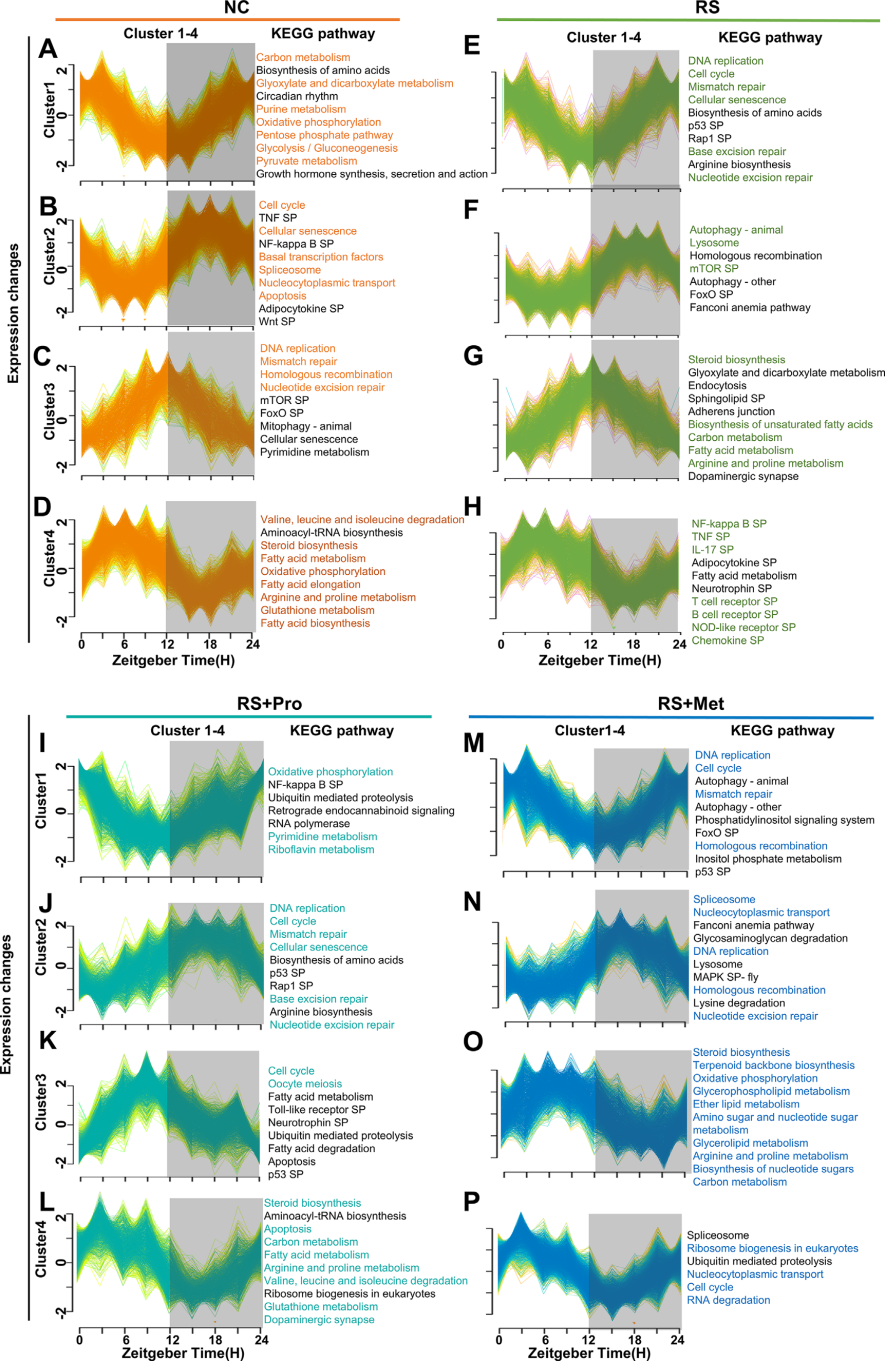
**Temporal clustering and KEGG pathway enrichment analysis of rhythmic genes in mouse corneal tissue under different treatment conditions.** **(A–P)** Time-series clustering analysis of rhythmic gene expression in the corneas of NC **(A–D)**, RS **(E–H)**, RS + Pro **(I–L)**, and RS + Met **(M–P)** groups. Rhythmic genes were grouped into four clusters (Cluster 1–4) based on their expression dynamics over a 24-hour period. The y-axis represents Z-score–normalized expression; the x-axis represents ZT0–ZT24. Each colored line represents the temporal expression trajectory of an individual gene. *Gray shading* indicates the dark phase (ZT12–ZT24). **(E–H)** KEGG pathway enrichment results (*q* < 0.05) for each gene cluster in the RS group. **(I–L)** KEGG pathway enrichment results (*q* < 0.05) for each gene cluster in the RS + Pro group. **(M–P)** KEGG pathway enrichment results (*q* < 0.05) for each gene cluster in the RS + Met group. Enriched pathways are listed to the right of each cluster. Pathway text colors denote the originating group: *orange* (NC), *green* (RS), *teal* (RS + Pro), and *blue* (RS + Met).

Having established these differential pharmacodynamic effects on circadian organization and immune pathways, we next investigated how chronic stress reshapes the temporal expression trajectories of rhythmic genes through time-series clustering analysis. To examine how RS reshapes the temporal expression and functional repertoires of CRGs, we combined time-series clustering with KEGG enrichment analysis. Z-score-normalized rhythmic profiles identified by JTK_CYCLE were partitioned into four biologically coherent clusters (Clusters 1–4) selected to maximize interpretability and statistical robustness (Figs. 5A–P).

Cluster 1 CRGs showed reproducible peaks at ZT3 and ZT21 and minima at ZT12 in all groups (Figs. 5A, 5E, 5I, 5M). In NC corneas these genes were chiefly associated with basal metabolic pathways (*q* < 0.05). RS shifted the same gene set toward pathways linked to cell-proliferation and tissue-repair processes. After propranolol treatment, enrichment was redirected to oxidative phosphorylation and pyrimidine metabolism, whereas metyrapone largely preserved the RS-type profile, with enrichment in DNA-replication and mismatch-repair pathways.

Cluster 2 CRGs displayed peaks at ZT18 and troughs at ZT6 (Figs. 5B, 5F, 5J, 5N). In NC tissue they were enriched for cell-cycle and general signaling pathways. RS introduced significant enrichment for lysosome, mTOR and autophagy pathways, indicative of stress-adaptive responses. Propranolol shifted enrichment back toward proliferation- and signaling-related pathways, whereas metyrapone retained spliceosome and DNA-replication enrichment patterns similar to RS.

Cluster 3 genes peaked at ZT12 in NC, RS and RS + Pro groups but shifted to ZT6 in RS + Met samples (Figs. 5C, 5G, 5K, 5O). In NC corneas they were mainly linked to cell-cycle regulation. RS redirected enrichment toward fatty-acid biosynthesis. Propranolol broadened the spectrum to include both cell-cycle and metabolic pathways, whereas metyrapone maintained a primary focus on metabolic processes.

Cluster 4 genes showed a uniform rhythm across all groups, peaking at ZT3 and reaching a trough at ZT18. In NC corneas, these transcripts were predominantly enriched in core metabolic pathways (Fig. 5D). RS redirected the same gene set toward immune-related pathways, including IL-17 signaling, NOD-like receptor signaling and other adaptive-immune processes (Fig. 5H). This immune enrichment was notably reduced in both the RS + Pro and RS + Met groups (Figs. 5L, 5P).

Collectively, these data demonstrate that chronic stress significantly remodels the circadian expression dynamics and temporal pathway enrichments of corneal CRGs. Pharmacological interventions differentially restored these patterns, indicating distinct modulatory roles of sympathetic and glucocorticoid pathways in circadian regulatory networks.

### Chronic Stress Preserves the Oscillatory Architecture of Core Clock Genes in the Cornea but Alters Temporal Expression at Specific Time Points

**Figure 6.**
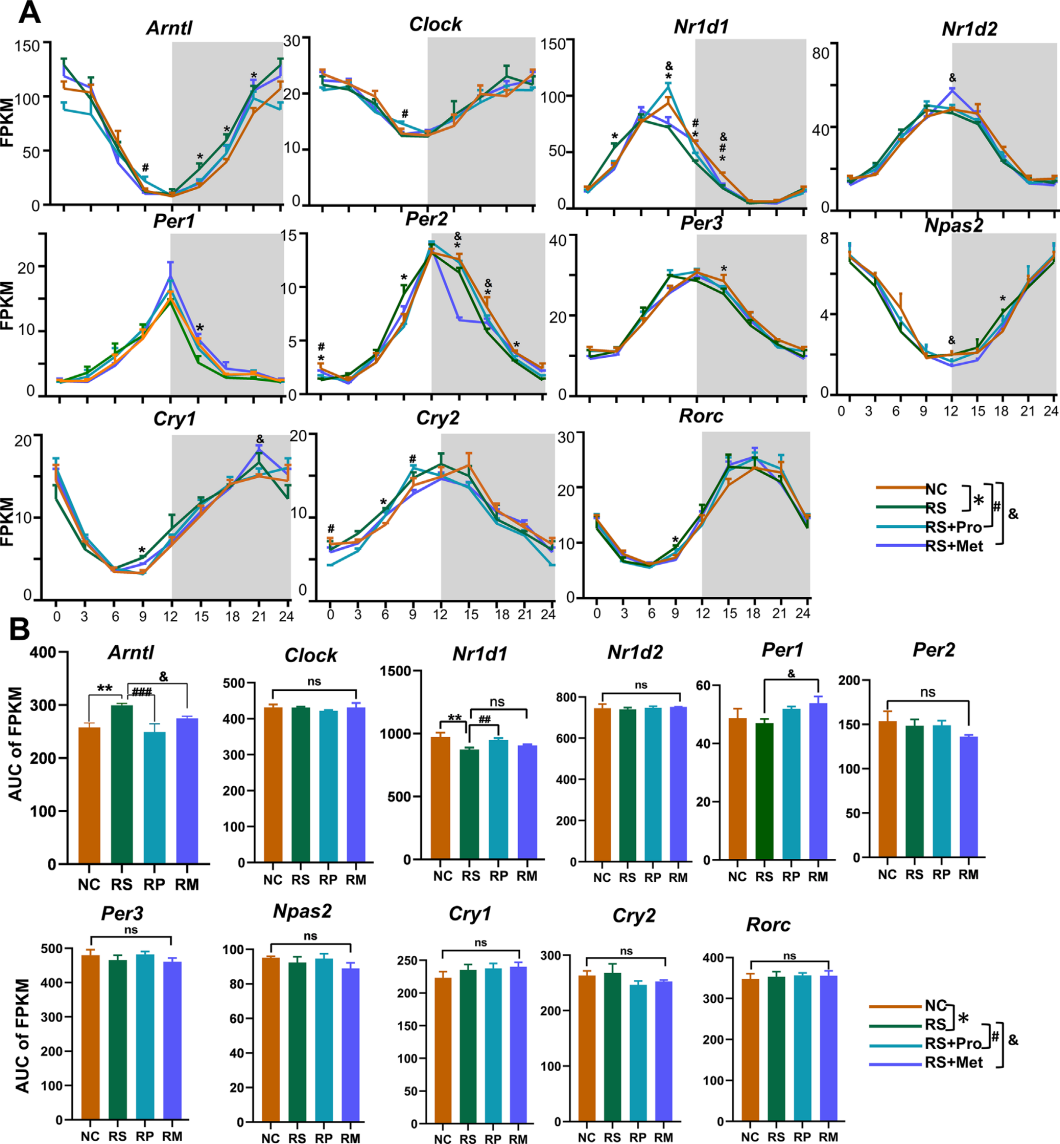
**Temporal expression patterns of core circadian clock genes in the cornea under chronic stress and pharmacological interventions.** **(A)** Expression profiles of 11 core circadian clock genes—*Arntl*, *Clock*, *Nr1d1*, *Nr1d2*, *Per1, Per2, Per3, Npas2, Cry1, Cry2*, and *Rorc*—in the corneas of mice under NC, RS, RS+Pro, and RS+Met conditions. Gene expression was measured as FPKM across eight ZT points (ZT0–ZT21), sampled every three hours (*n* = 3 per time point). *Gray-shaded areas* indicate the dark phase (ZT12–ZT24). Data are presented as mean ± SEM. Statistical comparisons at individual time points were evaluated using adjusted *P* values (*P < 0.05 between NC and RS group, #*P* < 0.05 between NC and RS + Pro group, &*P* < 0.05 between NC and RS + Met group). **(B)** Area under the curve analysis of 24-hour expression profiles for the 11 circadian clock genes. Y-axis represents cumulative FPKM expression. Data are shown as mean ± SEM. Statistical comparisons across groups were indicated using adjusted *P* values (&*P* < 0.05 between NC and RS + Met group, **P < 0.01 between NC and RS group, ##*P* < 0.01 between NC and RS + Pro group, ###*P* < 0.001 between NC and RS + Pro group; ns, not significant).

We next examined whether these transcriptional alterations were accompanied by corresponding changes in the core molecular clock machinery. To determine whether RS alters core circadian regulation in the cornea, we analyzed the temporal expression profiles of 11 canonical clock genes—*Arntl, Clock, Nr1d1, Nr1d2, Per1, Per2, Per3, Npas2, Cry1, Cry2,* and *Rorc*. Corneas were harvested every three hours from ZT0 to ZT21, covering an entire circadian cycle, in the NC, RS, RS + Pro, and RS + Met groups (Fig. 6A).

All 11 clock genes remained significantly rhythmic in every group, indicating that RS did not abolish the core circadian oscillator. Relative to NC, however, RS altered the temporal expression of nine genes—*Arntl, Nr1d1, Per1, Per2, Per3, Npas2, Cry1, Cry2,* and *Rorc*—at one or more Zeitgeber times (*P* < 0.05). In particular, *Arntl, Nr1d1,* and *Per2* showed persistently lower expression at several time points, consistent with phase misalignment accompanied by dampened amplitude.

Pharmacological treatments partially restored circadian gene expression patterns. Both propranolol and metyrapone significantly improved expression profiles for *Arntl, Nr1d1, Per1, Per3, Npas2*, and *Rorc*. Nonetheless, expression trajectories did not fully return to NC levels, indicating partial correction and a potential influence of multi-factorial regulation on corneal clock gene stability under stress.

To quantify changes in circadian output, the area under the curve over 24 hours was calculated for each gene (Fig. 6B). Significant differences were detected for *Arntl* and *Nr1d1* between RS and NC groups (*P* < 0.05). *Arntl* expression was significantly reduced in both RS + Pro and RS + Met groups, whereas *Nr1d1* was significantly elevated in RS + Pro and showed a nonsignificant upward trend in RS + Met.

These results indicate that chronic stress perturbs the phase and amplitude of several core clock genes while leaving their overall rhythmicity intact. The partial rescue by propranolol versus metyrapone further suggests that sympathetic signaling and glucocorticoid signaling make distinct contributions to preserving corneal circadian organization under stress.

### Chronic Stress Alters Neural Gene Expression and Impairs Corneal Innervation, Partially Alleviated by Pharmacological Intervention

**Figure 7.**
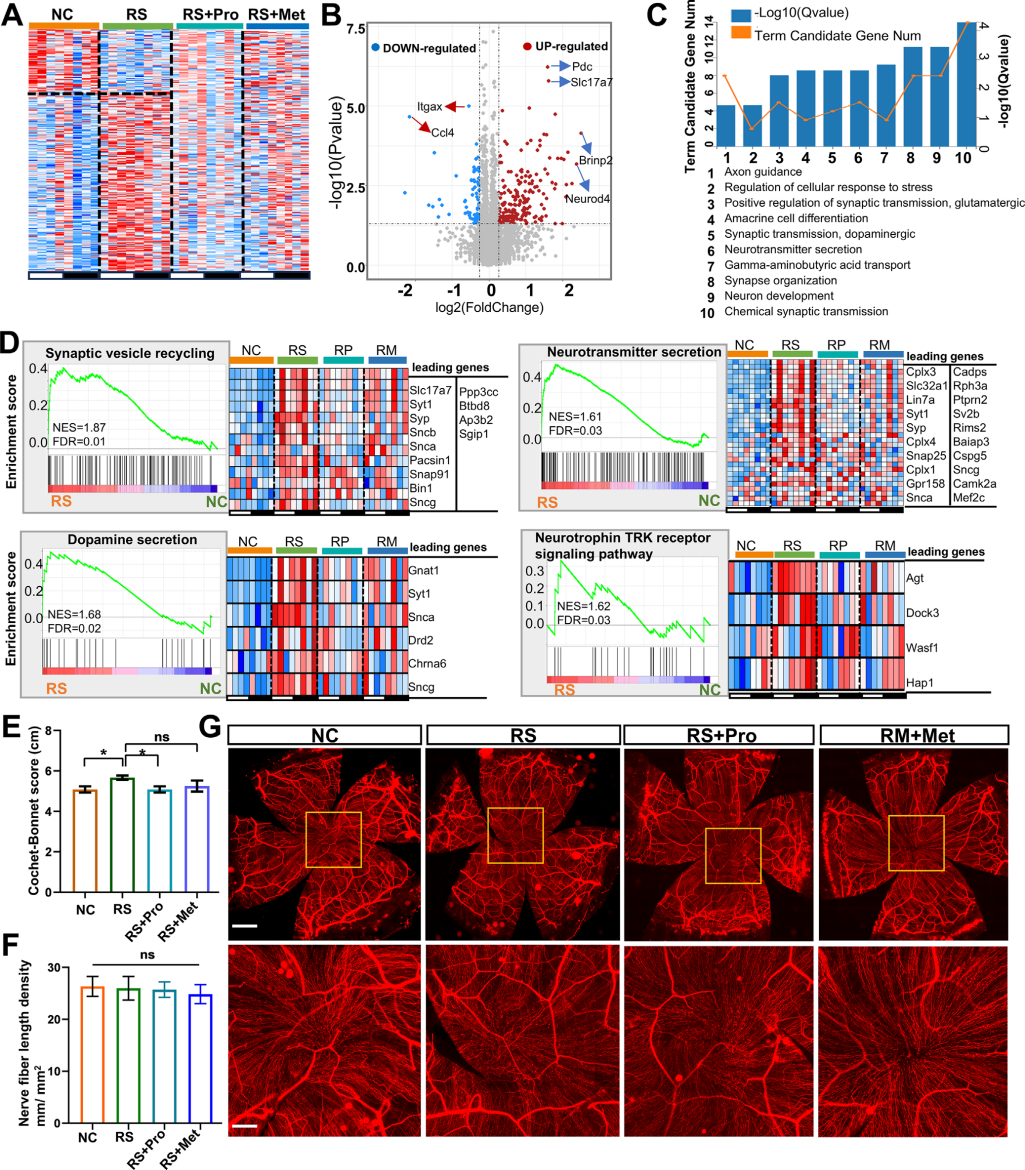
**Neural-related transcriptional changes and neuro-structural measurements in mouse cornea.** **(A)** Heat-map of neural-associated genes at eight Zeitgeber times (ZT0–ZT24) in NC, RS, RS + Pro and RS + Met groups. **Red**, higher expression; *blue*, lower expression. **(B)** Volcano plot of differentially expressed neural genes in RS versus NC corneas (|log₂FC| ≥ 0.263, *P* < 0.05). *Red* and *blue dots* denote up- and downregulated genes; selected genes are annotated. **(C)** GO enrichment of neural DEGs. *Blue bars* indicate –log₁₀(q); the overlaid *orange line* shows gene counts per term. **(D)** GSEA for four neural pathways: synaptic-vesicle recycling, neurotransmitter secretion, dopamine secretion and neurotrophin-TRK-receptor signaling. *Left panels* list normalized NES and FDR; *right panels* display heat-maps of leading-edge gene expression across groups. **(E)** Corneal mechanical sensitivity measured with a Cochet-Bonnet esthesiometer (mean ± SEM, *n* = 6). One-way ANOVA with Bonferroni correction; *P < 0.05. **(F)** Total length of βIII-tubulin–positive nerve fibers per unit area in the central cornea (mean ± SEM, n = 4); ns, not significant. **(G)** Whole-mount images of corneal innervation stained with NL557-conjugated rabbit anti-βIII-tubulin antibody. *Overview scale bar*: 300 µm; *inset scale bar*: 100 µm.

Having established the extent of core clock perturbation, subsequent analyses focused on downstream functional domains—namely, neural regulation, immune homeostasis, and epithelial–metabolic coupling. To examine the impact of chronic stress on corneal neurofunctional regulation, we performed transcriptomic profiling of neural-related genes in corneal tissues from NC, RS, RS + Pro, and RS + Met groups. In the RS group, a total of 198 neural-associated genes were significantly upregulated and 65 were downregulated (|log₂FC| > 0.263, *P* < 0.05), indicating stress-induced transcriptional remodeling. Heatmap comparisons showed that both propranolol and metyrapone treatment groups exhibited partial reversal of these expression changes (Fig. 7A).

Volcano plot analysis revealed several significantly upregulated genes in the RS group associated with neurotransmitter metabolism and synaptic function, including *Pdc*, *Slc17a7*, *Brinp2*, and *Neurod4* (log₂FC > 1.5, *P* < 0.05) (Fig. 7B). GO enrichment analysis showed that differentially expressed neural genes were enriched in pathways related to neurotransmitter secretion, dopaminergic synaptic transmission, synapse organization, and neuronal development (*q* < 0.05) (Fig. 7C).

GSEA further confirmed positive enrichment of neural pathways in the RS group, including synaptic vesicle recycling (NES = 1.87, FDR = 0.01), dopamine secretion (NES = 1.68, FDR = 0.02), and neurotrophin TRK receptor signaling (NES = 1.62, FDR = 0.03) (Fig. 7D). Heatmaps of representative genes from these pathways showed that expression trends in RS + Pro and RS + Met groups were partially restored toward control levels, especially in genes related to synaptic vesicle dynamics and dopaminergic function.

The corneal sensitivity of all four groups of mice was assessed using a Cochet-Bonnet aesthesiometer. The results showed that, compared with the NC group, the mechanical sensitivity of the RS group was significantly increased (*P* < 0.05). Propranolol treatment was able to restore corneal responsiveness to a certain extent (*P* < 0.05), whereas the effect of metyrapone treatment was not significant (Fig. 7E). In addition, there was no significant difference in the density of βIII-tubulin-positive nerve fibers in the central cornea among the groups (Figs. 7F, 7G). Because sympathetic activity is a key arm of the stress response, we asked whether sympathetic axons contribute to the corneal plexus. Tyrosine-hydroxylase/βIII-tubulin double immunofluorescence identified sparse but reproducible TH^+^/βIII-tubulin^+^ fibers in the limbal stroma (Supplementary Fig. S2). These fibers corroborate our earlier evidence of functional sympathetic innervation in the peripheral cornea and its role in wound repair during sleep deprivation.^19^^,^^47^ Taken together, RS compromises corneal innervation and sensory function, and these deficits can be mitigated—though not fully reversed—by targeting either β-adrenergic or glucocorticoid signaling pathways.

### RS Suppresses Immune Gene Expression and Disrupts the Rhythmic Recruitment of Immune Cells

**Figure 8.**
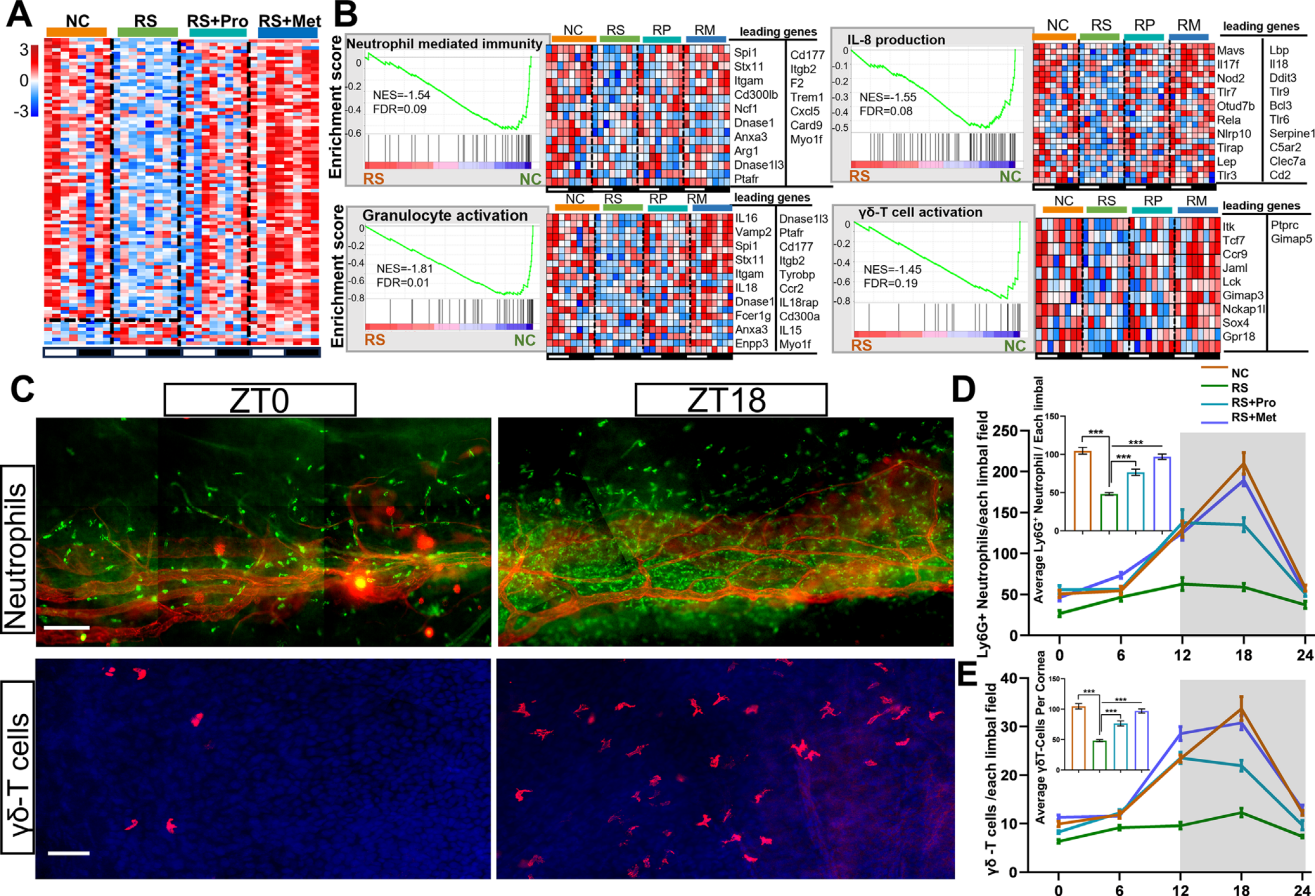
**Comparative analysis of corneal immune gene expression and immune cell profiles under stress and pharmacological intervention.** **(A)** Heatmap showing expression of immune-related genes across ZT points (ZT0–ZT21) in NC, RS, RS + Pro, and RS + Met groups. *Red* indicates high expression; *blue* indicates low expression. **(B)** GSEA comparing RS and NC groups identified negative enrichment in immune pathways, including neutrophil-mediated immunity, granulocyte activation, IL-8 production, and γδ-T cell activation. NES and FDR are shown. Heatmaps display expression of leading-edge genes across groups. **(C)** Representative immunofluorescence images of the NC group corneal tissue at ZT0 and ZT18, showing neutrophils (Ly6G, *green*) and blood vessels (CD31, *red*) (*Scale bar*: 50 µm), and γδ-T cells (TCRδ, *red*) (*Scale bar*: 20 µm). **(D)** Quantification of Ly6G⁺ neutrophils at ZT0, ZT6, ZT12, ZT18 and ZT24 (*n* = 6, mean ± SEM). The top left corner shows the mean number of neutrophils over the entire circadian cycle in each group. ***P < 0.001. **(E)** Quantification of γδ-TCR⁺ cells at ZT0, ZT6, ZT12, ZT18, and ZT24 (*n* = 6, mean ± SEM). The top left corner shows the mean number of γδ T cells over the entire circadian cycle in each group. ***P < 0.001.

To evaluate the effects of RS on corneal immune homeostasis, we examined the transcriptional profiles of immune-related genes and inferred immune-cell infiltration across groups. In the RS corneas, immune-associated transcripts were broadly downregulated, whereas both propranolol and metyrapone treatments partially restored their expression (Fig. 8A).

GSEA further indicated negative enrichment of multiple immune-related pathways in the RS group, including neutrophil-mediated immunity (NES = −1.54, FDR = 0.09), IL-8 production (NES = −1.55, FDR = 0.08), granulocyte activation (NES = −1.81, FDR = 0.01), and γδ-T cell activation (NES = −1.45, FDR = 0.19; Fig. 8B). Key immune genes such as *Spi1, Il16,* and *Il18* showed increased expression in both intervention groups, suggesting partial reversal of stress-induced immunosuppression.

Immunofluorescence analysis revealed a significant reduction in corneal immune-cell abundance in RS-treated mice (*P* < 0.001). Immune-cell counts were partially restored after pharmacologic intervention with either propranolol or metyrapone (Figs. 8C–E). To assess whether immune-cell recruitment to the cornea is under circadian control, we quantified immune cells at five circadian time points (ZT0, ZT6, ZT12, ZT18, ZT24) and evaluated rhythmicity using the JTK_CYCLE algorithm. Significant circadian oscillations were detected in the NC group, as well as in both intervention groups (RS + Pro and RS + Met; *q* < 0.05). In contrast, rhythmicity was abolished in the RS group, indicating that chronic stress disrupts the circadian regulation of corneal immune-cell dynamics (Figs. 8D, 8E).

Taken together, these findings demonstrate the suppression of multiple immune-related gene sets and the rewiring of immune signaling pathways, accompanied by a loss of temporal coordination. Pharmacological blockade of β-adrenergic or glucocorticoid signaling partially reversed these deficits, with metyrapone exhibiting a stronger capacity to restore immune-related transcriptional activity.

### RS Induces Metabolic Reprogramming and Enhances Energy-Associated Transcriptional Activity in the Cornea

**Figure 9.**
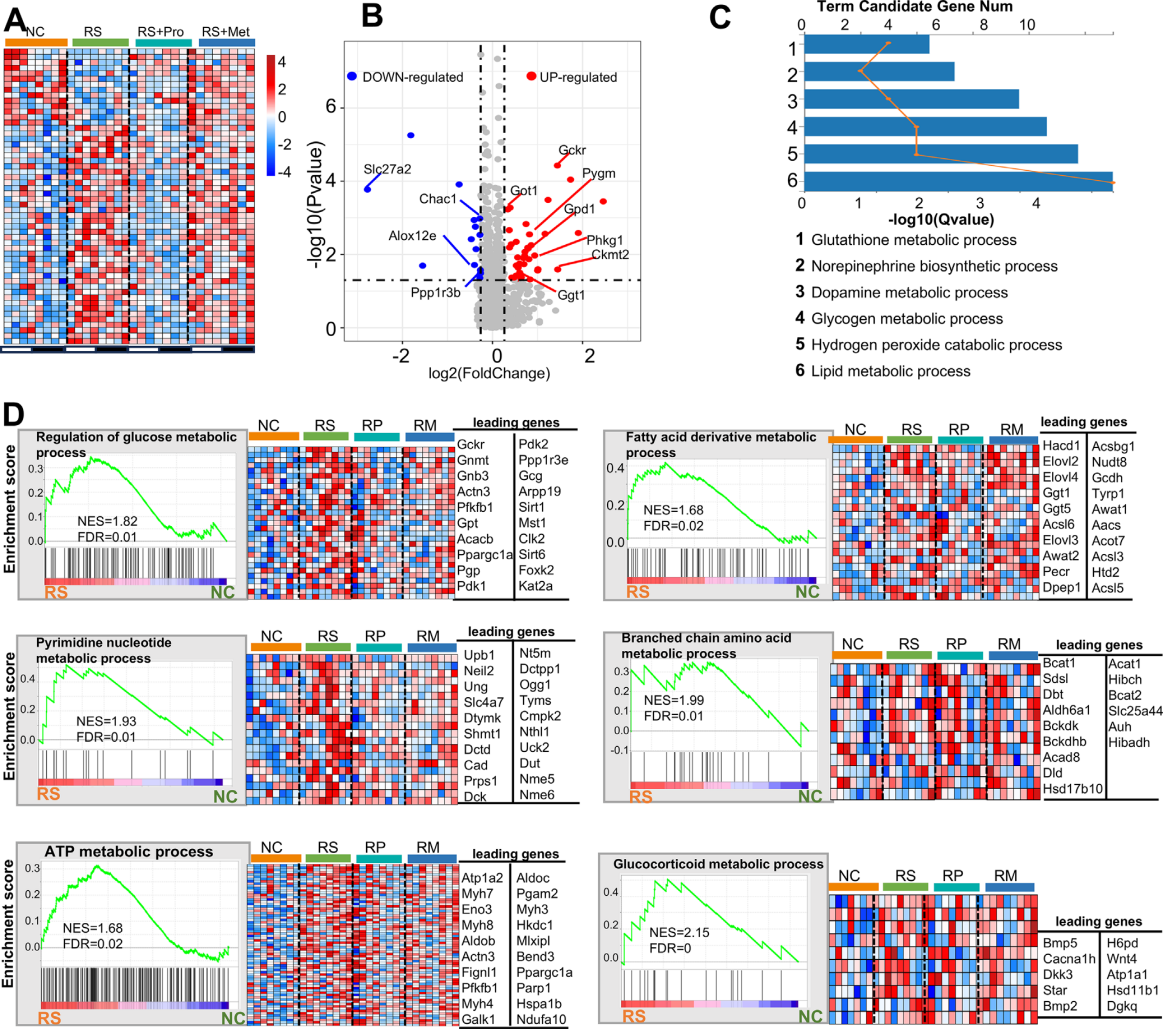
**Comparative analysis of corneal metabolic transcriptomic features under stress and pharmacological intervention.** **(A)** Heatmap showing expression profiles of metabolism-related genes across ZT points (ZT0–ZT21) in NC, RS, RS + Pro, and RS + Met groups. *Red* indicates high expression; *blue* indicates low expression. **(B)** Volcano plot of differentially expressed metabolic genes between NC and RS groups. *Red* and *blue dots* indicate significantly upregulated and downregulated genes, respectively (|log₂FC| > 0.263, *P* < 0.05). A total of 40 upregulated and 14 downregulated genes were identified. **(C)** GO enrichment analysis of DEGs showing significantly enriched metabolic pathways (*q* < 0.05). The x-axis indicates −log₁₀ (*q* value), and the y-axis lists the enriched biological processes. **(D)** GSEA of metabolism-related pathways in RS versus NC groups. Each panel displays the NES and FDR. Adjacent heatmaps show expression profiles of leading-edge genes across the four groups. If the number of leading genes exceeded 20, the top 20 genes were shown.

To characterize metabolic reprogramming after RS, we interrogated metabolism-associated transcripts in corneas from NC, RS, RS + Pro and RS + Met mice. Compared with NC, the RS group showed differential expression of 54 metabolic genes—40 upregulated and 14 downregulated (|log₂FC| ≥ 0.263, *P* < 0.05; Fig. 9A). Upregulated genes included regulators of glucose homeostasis such as *Gckr* (*P* < 0.05), suggesting activation of glycolytic pathways under stress (Fig. 9B). GO enrichment indicated significant over-representation of glycogen and lipid metabolism, glutathione metabolism, hydrogen-peroxide catabolism, and catecholamine (dopamine, NE) biosynthesis pathways (*q* < 0.05; Fig. 9C), consistent with increased energetic demand and oxidative-stress buffering in stressed corneas.

GSEA further demonstrated positive enrichment of key energy-related pathways in the RS group, including regulation of glucose metabolism (NES = 1.82, FDR = 0.01), ATP metabolic process (NES = 1.68, FDR = 0.02), pyrimidine nucleotide metabolism (NES = 1.93, FDR = 0.01), branched-chain amino acid metabolism (NES = 1.99, FDR = 0.01), glutathione metabolism, and vitamin metabolic pathways (NES = 2.15, FDR = 0; Fig. 9D). Heatmaps of leading-edge genes showed that propranolol treatment partially reversed gene expression in several of these pathways, most notably glucose and ATP metabolism. In contrast, metyrapone treatment yielded limited restoration, with only modest recovery observed in selected pathways such as pyrimidine nucleotide metabolism.

These data indicate that RS provokes a distinct metabolic reprogramming in the cornea, marked by heightened glucose- and amino-acid-metabolic activity together with reinforced oxidative-stress defenses. The more complete normalization achieved with propranolol implies that sympathetic signaling is the principal driver of these stress-related metabolic alterations.

### Chronic Stress Alters Corneal Epithelial Proliferation and Barrier-Associated Features

**Figure 10.**
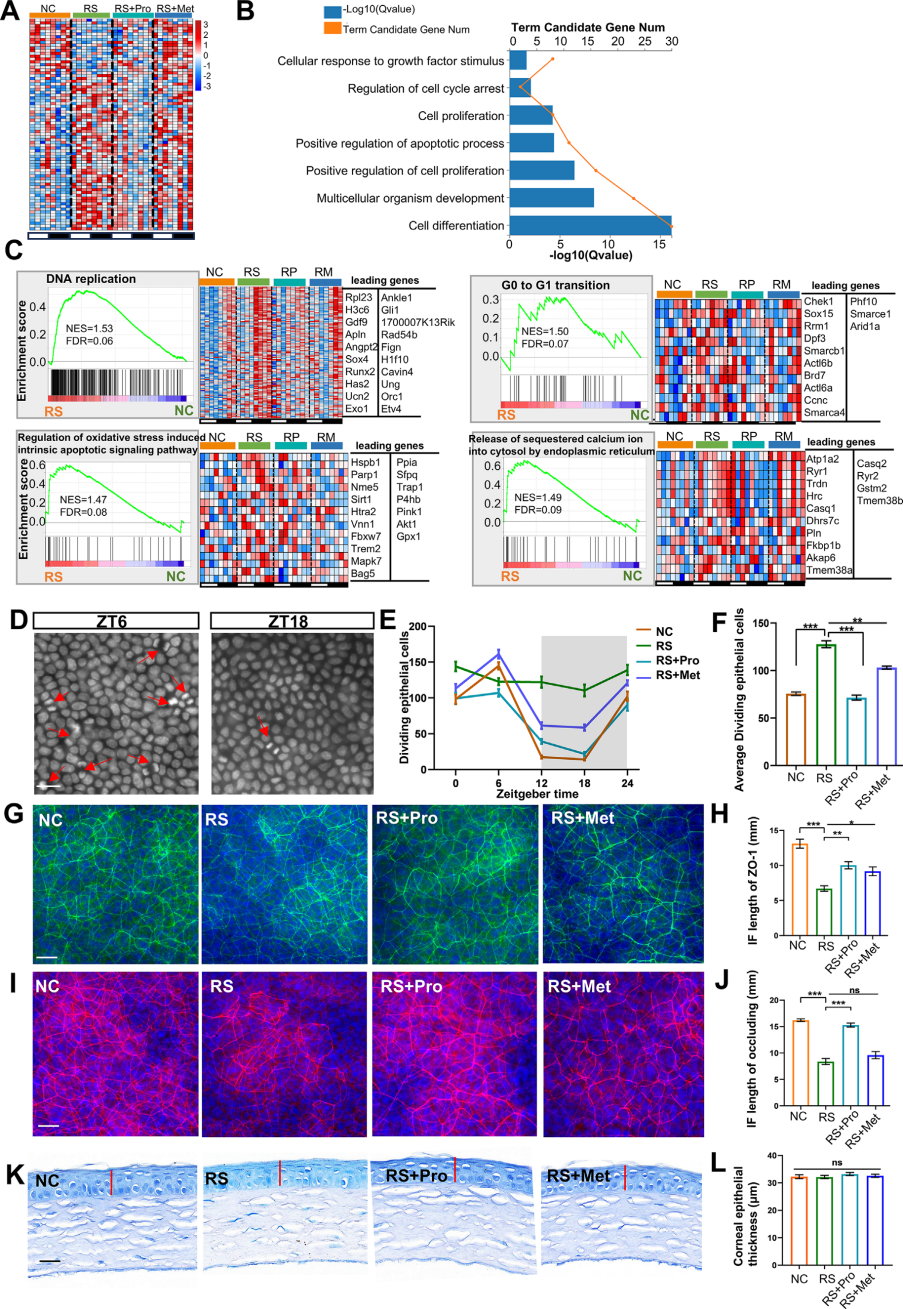
**Comparative analysis of corneal epithelial cell cycle and barrier-related features under stress and pharmacological intervention.** **(A)** Heatmap showing expression levels of cell cycle–related genes across ZT points (ZT0–ZT21) in NC, RS, RS + Pro, and RS + Met groups. *Red* indicates high expression, and *blue* indicates low expression. **(B)** GO enrichment analysis of DEGs, showing enrichment in biological processes related to cell proliferation and cycle regulation. The x-axis indicates −log₁₀ (*q* value). **(C)** GSEA analysis indicating significant enrichment in pathways including DNA replication, G0 to G1 transition, regulation of oxidative stress induced intrinsic apoptotic signaling pathway, and release of sequestered calcium ion into cytosol by endoplasmic reticulum in RS group. NES and FDR are provided. Adjacent heatmaps show expression of leading-edge genes across all groups. **(D–F)** Representative images **(D**; *scale bar*: 30 µm) and quantification **(E**; *n* = 6, mean ± SEM, ***P < 0.001, **P < 0.01) of mitotic corneal epithelial cells with paired nuclei at ZT0, ZT6, ZT12, ZT18, ZT24. **(G, H)** Representative ZO-1 immunofluorescence images **(G)** and quantitative comparison of fluorescence signal per unit length **(H**; *n* = 6, mean ± SEM, *P < 0.05, **P < 0.01, ***P < 0.001) in corneal epithelium across groups. **(I, J)** Representative images of occludin expression **(I**; *scale bar*: 30 µm) and corresponding fluorescence quantification **(J**; *n* = 6, mean ± SEM, ***P < 0.001, ns, not significant). **(K, L)** Toluidine blue–stained central corneal sections **(K**; *scale bar*: 30 µm) and measurement of epithelial thickness under ×40 magnification **(L**; *n* = 6, mean ± SEM, ns, not significant).

To examine how RS affects corneal epithelial renewal and barrier integrity, we profiled cell-cycle and proliferation-related transcripts and assessed barrier proteins ZO-1 and occludin by immunofluorescence. Differential-expression analysis identified 76 cell-cycle/proliferation genes that were significantly altered in RS versus NC corneas (|log₂FC| ≥ 0.263, *P* < 0.05): 56 were upregulated and 20 downregulated (Fig. 10A).

GO enrichment analysis indicated that the DEGs were significantly enriched in biological processes including regulation of cell cycle arrest, cell proliferation, and cell differentiation (Fig. 10B). GSEA further showed significant positive enrichment of cell cycle–associated pathways in the RS group (Fig. 10C), particularly DNA replication (NES = 1.53, FDR = 0.06) and G0 to G1 transition (NES = 1.50, FDR = 0.07). Meanwhile, pathways related to apoptosis were also significantly and positively enriched, such as the Regulation of oxidative stress-induced intrinsic apoptotic signaling pathway (NES = 1.47, FDR = 0.08) and the release of sequestered calcium ion into cytosol by endoplasmic reticulum (NES = 1.49, FDR = 0.09). Heatmaps of representative genes demonstrated that propranolol and metyrapone treatments partially attenuated stress-induced transcriptional changes, especially in DNA replication–related modules.

Time-series analysis of corneal epithelial cell proliferation demonstrated that RS significantly disrupted the circadian rhythm of epithelial mitosis (*q* < 0.05) and led to a significant increase in mitotic cell numbers over the 24-hour cycle (*P* < 0.05). Pharmacological intervention with either metyrapone or propranolol partially restored rhythmic fluctuations in mitotic activity and reduced overall mitotic burden relative to the RS group (Figs. 10D–F).

Quantitative immunofluorescence analysis showed that fluorescence intensity per unit length of the tight junction proteins ZO-1 and occludin was reduced by approximately 50% in RS corneas (*P* < 0.01). Propranolol partially restored junctional protein expression (*P* < 0.05), whereas metyrapone had minimal effect (Figs. 10G–J). Central epithelial thickness did not differ significantly among groups (Figs. 10K, L), indicating that the hyperproliferative response occurred in the absence of overt epithelial thinning. To preliminarily evaluate the barrier function of the corneal epithelium, fluorescein sodium staining was performed. The results demonstrated no detectable punctate fluorescein sodium staining in the corneal epithelium across all four experimental mouse groups (Supplementary Fig. S3).

Collectively, these findings indicate that RS disrupts the circadian regulation of corneal epithelial turnover and promotes hyperproliferation, while downregulating key barrier proteins. These alterations likely reflect a compensatory epithelial response to neuroendocrine stress. Both β-adrenergic blockade and glucocorticoid synthesis inhibition mitigated these effects, with propranolol demonstrating greater efficacy—supporting a primary role for sympathetic signaling in stress-induced epithelial and barrier dysregulation.

## Discussion

The present study shows that RS misaligns the corneal circadian transcriptome and is associated with changes in neural, immune, metabolic and epithelial readouts. Pharmacologic attenuation of β-adrenergic or glucocorticoid signaling corrected discrete subsets of these alterations, indicating complementary—but non-redundant—roles in ocular-surface regulation.

Under physiological conditions, the corneal clock synchronizes DNA replication, purine biosynthesis, and barrier renewal, aligning mitotic peaks with periods of minimal environmental exposure.^17^^,^^18^^,^^40^^,^^50^ RS advanced the phase of approximately half of rhythmically expressed corneal genes and dampened their amplitudes, while core clock genes (e.g., *Arntl, Per2, Cry1*) retained rhythmicity. Similar stress-associated shifts that spare core oscillators but alter peripheral outputs have been described in liver, heart, and hippocampus.^51^^–^^55^ Propranolol partially restored phase alignment, whereas metyrapone produced a more modest effect, consistent with evidence that catecholamines and glucocorticoids can reset peripheral clocks.^56^^,^^57^ Collectively, these findings support the cornea as a stress-sensitive peripheral oscillator in which neuroendocrine signals act chiefly on downstream clock-controlled outputs rather than the core loop.^16^

RS enhanced corneal mechanical sensitivity, and both propranolol and metyrapone restored responses toward control levels. By contrast, the areal length of βIII-tubulin–positive fibers in the central cornea did not differ among groups. These findings indicate a predominantly functional change in corneal sensation without detectable loss of sub-basal nerve architecture, which is in line with prior rodent studies reporting stress-related alterations in corneal mechanical sensitivity.^58^

TH/βIII-tubulin staining revealed sparse sympathetic-like axons at the limbus, indicating the presence of peripheral sympathetic input. Chronic sympathetic activation may modulate corneal sensation via regulation of neurotrophic factors (e.g., NGF, BDNF) and pro-inflammatory cytokines (e.g., IL-17, TNF-α),^59^ a possibility that warrants cornea-specific mechanistic testing.

RS broadly downregulated immune-related transcripts, including genes involved in neutrophil chemotaxis, Th17 signaling, and antigen presentation, consistent with stress-associated immunosuppression documented in other systems.^60^^–^^65^ Metyrapone partially restored immune-gene expression, aligning with glucocorticoid receptor–dependent modulation of immune rhythmicity via known interactions between the core clock machinery (e.g., *BMAL1*) and inflammatory regulators such as NF-κB.^56^ Disruption of immune circadian rhythms may impair the temporal coordination between corneal immune defenses and environmental exposures, diminishing protective responsiveness.

Metabolically, RS increased enrichment of glycolysis, tricarboxylic-acid-cycle, nucleotide-biosynthetic, and glutathione-metabolic pathways (*q* < 0.05), consistent with elevated energetic and redox demands and comparable to stress-related changes described in the brain.^66^^–^^69^ These alterations were only partially normalized by either treatment, suggesting additional regulators beyond β-adrenergic and glucocorticoid signaling.

Epithelial proliferation increased at ZT6 under RS, and transcriptomic signatures indicated reprogramming of cell-cycle rhythmicity, in line with reports that circadian disruption perturbs epithelial repair in other tissues.^70^^–^^73^ Propranolol partially corrected the proliferative change, whereas metyrapone showed limited effect.

Although the SNS and HPA pathways distinctly regulate corneal functions, they interact dynamically.^1^ For example, glucocorticoids acting through the glucocorticoid receptor can increase β₂-adrenergic-receptor expression and augment catecholaminergic capacity via PNMT induction, thereby enhancing sympathetic responsiveness.^74^^–^^76^ Glucocorticoids also reset peripheral circadian oscillators, providing a plausible route by which chronic stress amplifies local chronodisruption.^14^

Several limitations temper the interpretation of these findings. First, the murine cornea differs from human counterparts in stromal organization, thickness, and nerve density, potentially altering stress responses.^77^^,^^78^ Second, this study used only male mice, although sex-specific differences in corneal physiology and stress reactivity are recognized.^79^^–^^81^ Third, RS may indirectly affect the ocular surface by altering lacrimal and meibomian gland secretions and destabilizing the tear film—factors not measured here.^82^^–^^84^ Fourth, systemic pharmacologic interventions (propranolol, metyrapone) preclude clear separation of systemic versus local corneal actions; cornea-targeted delivery will be required to define local mechanisms.^85^^,^^86^ Fifth, all measurements in this study were conducted under a 12:12 light/dark cycle and were not validated under constant darkness conditions. Consequently, the observed circadian fluctuations may result from the combined effects of entrainment by the LD cycle and stress-induced neuroendocrine signaling. Future studies using a constant darkness protocol are essential to fully dissect the autonomous contribution of endogenous circadian mechanisms. Sixth, the mechanisms by which RS impairs corneal immune function were not explored in depth. RS may alter granulocyte/γδ T-cell recruitment via downregulation of epithelial chemokines such as *Cxcl5* and *Ccr9*; future work will integrate flow cytometry with temporal sampling to delineate immune-cell phenotypes and their circadian dynamics. Seventh, whole-tissue RNA-seq can obscure cellular heterogeneity; single-cell and spatial transcriptomics are needed to resolve cell-specific stress responses.^87^^,^^88^ Last, although current data demonstrate co-occurrence of circadian misalignment with epithelial, immune, and metabolic changes, directional causality cannot be inferred. There is also room for follow-up studies involving corneal epithelium-specific clock perturbations.^18^^,^^89^

**Figure 11.**
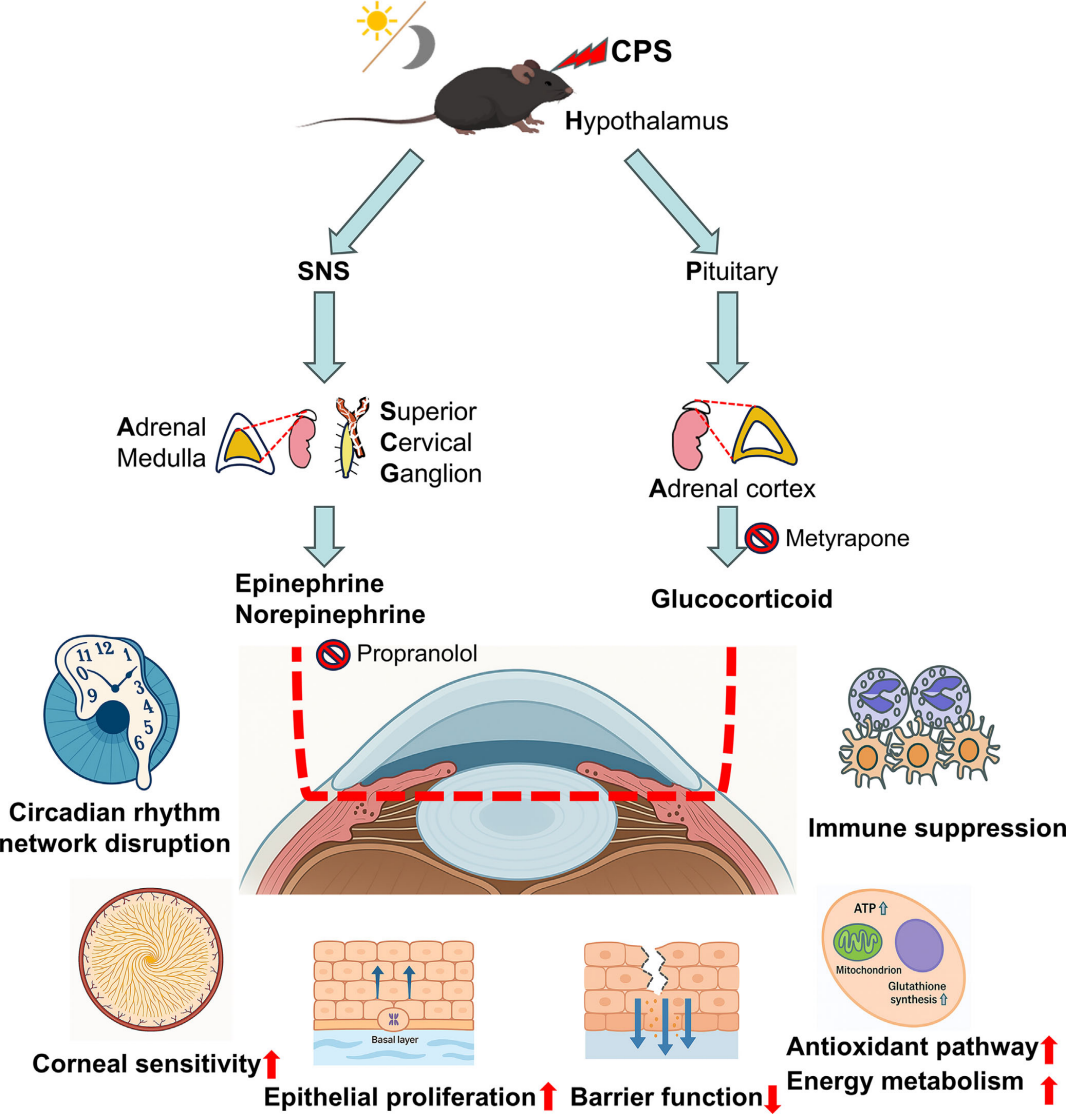
**RS misaligns the corneal clock and perturbs ocular-surface homeostasis via sympathetic and HPA-axis activation.** Schematic of the working model. RS activates the SNS and the HPA axis, increasing circulating catecholamines (EPI/NE) and glucocorticoids (corticosterone). These neuroendocrine signals impinge on the cornea to disrupt circadian timing (phase advance/amplitude dampening) and triggering functional changes across multiple domains: immune suppression (fewer neutrophils and γδ-T cells), metabolic/antioxidant upregulation (ATP and glutathione pathways), epithelial hyperproliferation (basal mitoses), barrier weakening (ZO-1/occludin↓), and enhanced corneal mechanical sensitivity. Pharmacologic modulation—β-adrenergic receptor blockade (e.g., propranolol) and inhibition of glucocorticoid synthesis (e.g., metyrapone)—partially attenuates clock misalignment and functional abnormalities, indicating distinct yet complementary roles of SNS and HPA signaling in stress-induced corneal reprogramming.

Notwithstanding these limitations, the present data support a working model wherein RS activates the SNS and the HPA axis, producing NE and glucocorticoids that disrupt the corneal circadian clock and impair structural, immune, neural, and metabolic functions (Fig. 11). Clinically, because several stress-induced defects exhibited drug- and circadian time–dependent responsiveness, chronotherapeutic strategies targeting β-adrenergic or glucocorticoid pathways—and, where appropriate, melatonin-based adjuncts—could enhance outcomes in stress-related ocular-surface disease (e.g., dry eye or postoperative healing).^90^

## Conclusions

Taken together, RS mis-times corneal circadian outputs—characterized by phase advance and qualitatively dampened amplitude—while altering epithelial proliferation, barrier markers, immune programs and metabolic pathways. Propranolol more effectively realigned phase than metyrapone, whereas both interventions provided partial functional rescue, consistent with distinct sympathetic and glucocorticoid contributions.

## Supplementary Material

Supplement 1
