# Supplementary material for: Chronic Restraint Stress Misaligns Corneal Clock via β-Adrenergic and Glucocorticoid Signaling, Altering Epithelial, Neural, Immune, Metabolic States
Source: Invest Ophthalmol Vis Sci. 2025 Dec 16;66(15):50. doi: 10.1167/iovs.66.15.50 (PMC12720190; doi:10.1167/iovs.66.15.50)
Supplement: Supplement 1 [file iovs-66-15-50_s001.docx]

**
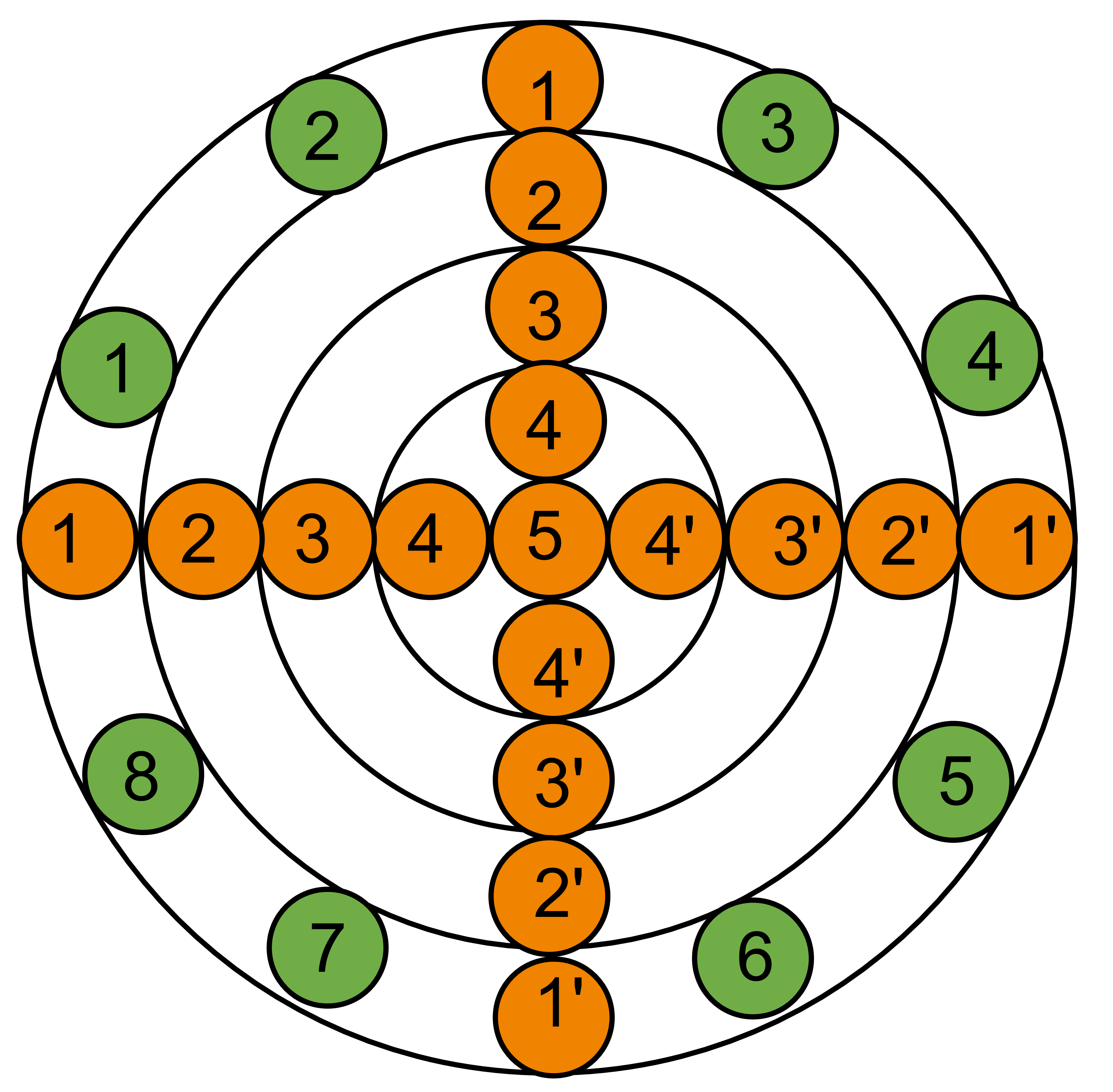
**

**Supplemental Figure 1.** Eight green circles represent 40× magnification fields used to quantify neutrophils surrounding limbal blood vessels. Orange fields labeled 1 to 5 and 5′ to 1′ are used for quantification of mitotic epithelial cells. Orange fields 1 to 3 are used for quantification of γδ-T cells.

**
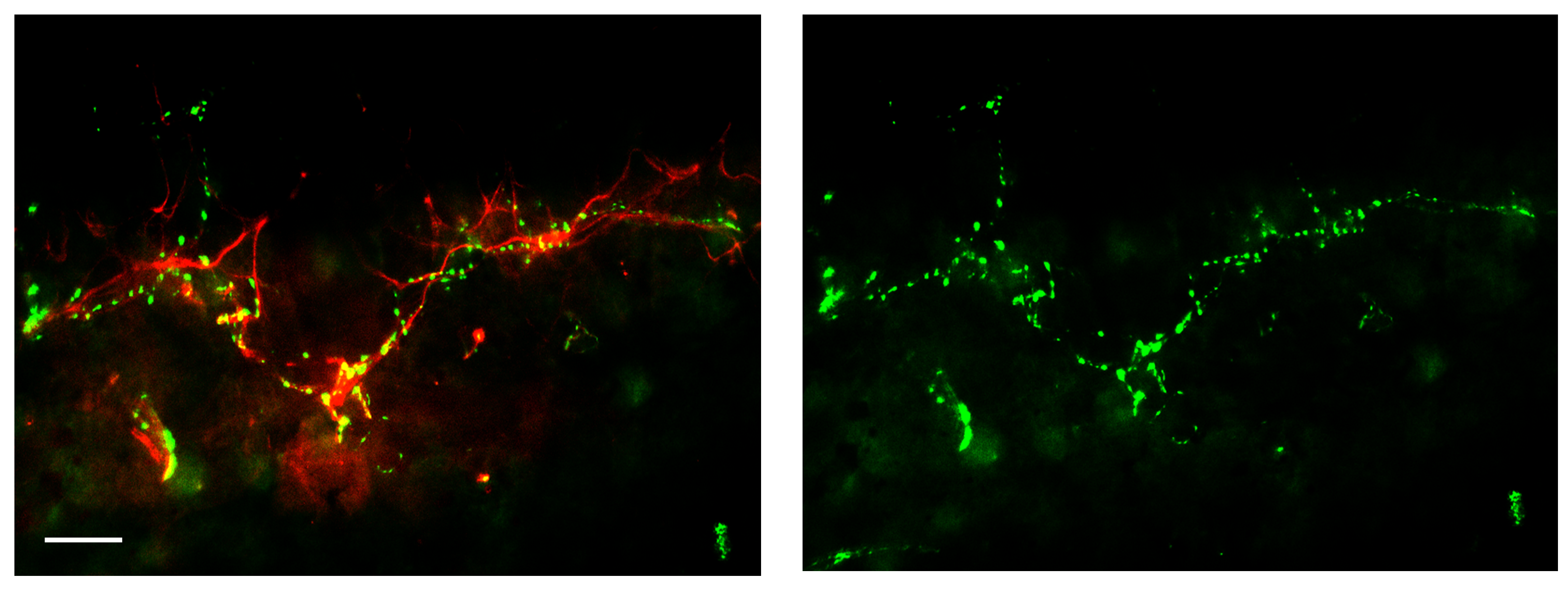
**

**Supplemental Figure 2.** Limbal nerve fibers co-stained for TH⁺ (green) and βIII-tubulin⁺ (red). Scale bar = 20 μm


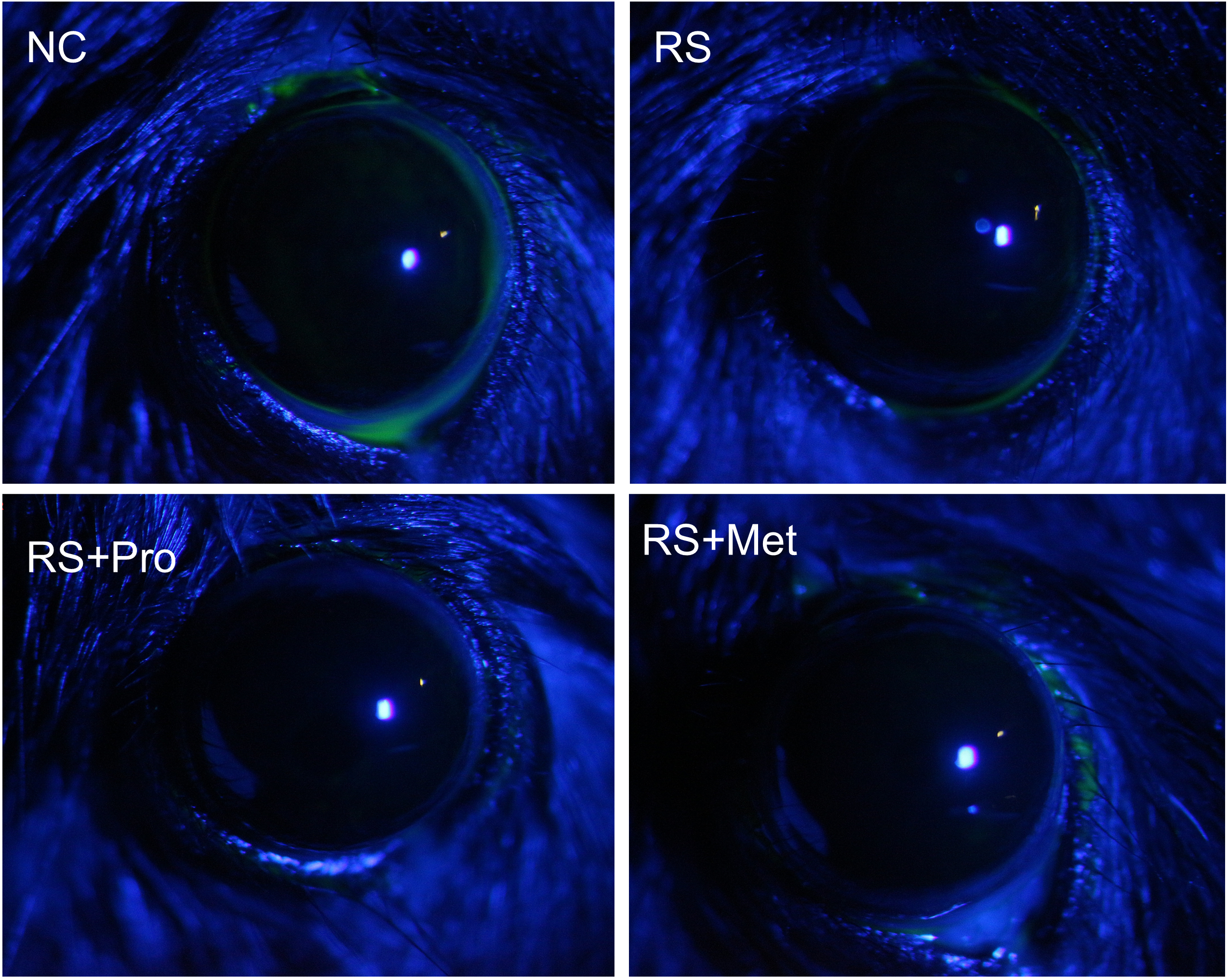


**Supplemental Figure 3.** Representative images of sodium fluorescein staining in the corneas of the four groups of mice.
